# Metal-based Heterogeneous Catalysts for One-Pot Synthesis of Secondary Anilines from Nitroarenes and Aldehydes

**DOI:** 10.3390/molecules26041120

**Published:** 2021-02-20

**Authors:** Giuseppe Romanazzi, Valentina Petrelli, Ambra Maria Fiore, Piero Mastrorilli, Maria Michela Dell’Anna

**Affiliations:** Dipartimento di Ingegneria Civile, Ambientale, del Territorio, Edile e di Chimica (DICATECh), Politecnico di Bari, via Orabona 4, Bari 70125, Italy; valentina.petrelli@poliba.it (V.P.); ambramaria.fiore@poliba.it (A.M.F.); p.mastrorilli@poliba.it (P.M.)

**Keywords:** reductive amination, secondary anilines, heterogenous catalysts, one-pot synthesis, metal nanoparticles, metal nanocomposites, tandem reactions

## Abstract

Recently, *N*-substituted anilines have been the object of increasing research interest in the field of organic chemistry due to their role as key intermediates for the synthesis of important compounds such as polymers, dyes, drugs, agrochemicals and pharmaceutical products. Among the various methods reported in literature for the formation of C–N bonds to access secondary anilines, the one-pot reductive amination of aldehydes with nitroarenes is the most interesting procedure, because it allows to obtain diverse *N*-substituted aryl amines by simple reduction of nitro compounds followed by condensation with aldehydes and subsequent reduction of the imine intermediates. These kinds of tandem reactions are generally catalyzed by transition metal-based catalysts, mainly potentially reusable metal nanoparticles. The rapid growth in the last years in the field of metal-based heterogeneous catalysts for the one-pot reductive amination of aldehydes with nitroarenes demands for a review on the state of the art with a special emphasis on the different kinds of metals used as catalysts and their recyclability features.

## 1. Introduction

### 1.1. General Considerations

Secondary amines occupy a paramount role as intermediates and building blocks for the production of numerous agrochemicals, detergents, drugs, dyes, herbicides, pharmaceuticals, pigments, and other fine chemicals [1,2]. From a synthetic point of view, it is mandatory to develop efficient, facile, sustainable, and low-cost preparations of secondary amines [3,4,5]. Among the most general synthetic approaches to obtain secondary amines, the amine-carbonyl reductive amination (RA) is by far the most preferable, because the substrates are readily available as well as the whole process occurs with a high atom-economy, putting RA in the essential toolboxes for both academic and industrial chemists [6,7,8,9,10,11,12,13,14]. RA is referred to as direct reductive amination (DRA), when the carbonyl compound, generally an aldehyde or a ketone, and the primary amine are mixed in the presence of the proper reducing agent without prior formation of the intermediate imine (or iminium salt if the amine is secondary). Otherwise, if the preformation of the intermediate imine is followed by reduction in a separate step, RA is referred to as indirect (or stepwise) reductive amination [15]. If the primary amines used in RA are anilines, nitroarenes can alternatively be employed as their precursors for accessing secondary amines, because anilines are easily achieved by the catalytic hydrogenation of nitroarenes [16,17,18]. In this case, a RA starting from a nitroarene is, in principle, more attractive because it avoids another hydrogenation step, saving time as well costs, since nitroarenes are cheaply available starting materials in organic synthesis [19]. Consequently, the one-pot reductive amination of aldehydes with nitroarenes is a very interesting procedure because it allows one to obtain diverse *N*-alkylated or *N*-benzylated anilines by simple reduction of nitro compounds, followed by condensation with aldehydes and subsequent reduction of the imine intermediates.

In the last two decades, several efforts have been addressed to develop an efficient heterogeneous catalytic system for this tandem reaction, that could be carried out in a direct way or stepwise, starting from nitroaromatics and aldehydes in the presence of supported metal catalysts, mainly nanostructured, or bimetals with a reducing agent. Indeed, nowadays, a central issue in contemporary synthetic organic chemistry is developing economically efficient and environmentally sustainable processes, and one of the best and widest approaches for reaching these objectives is the one-pot synthesis of a target molecule in the same reaction vessel, preferably facilitated by a recyclable heterogeneous catalyst [20,21]. The heterogeneous catalytic one-pot synthesis of secondary anilines by RA starting from nitroarenes and aldehydes is a clear example of this approach.

This mini-review has been conceived to critically summarize and discuss the developments of active ad selective metal or bimetal-based heterogeneous catalysts used in the one-pot synthesis of secondary anilines from nitroarenes and aromatic or aliphatic aldehydes with an appropriate reducing agent. The time horizon is the last two decades, but particular emphasis will be given to the intense activity emerged in the last luster, when the main players have become the catalysts based on the Earth-abundant 3 d metals, which have indeed replaced those based on noble metals. The discussion on the various catalysts has been focused also on the advantages of the catalysts in terms of recyclability without forgetting to underline some of their drawbacks. For highlights or reviews dealing with other aspects of reductive amination reactions for the synthesis of several kinds of amines, the reader is referred to the works reported in references [7,8,9,10,11,12,13,14,22,23,24].

### 1.2. Mechanism of the One-pot Synthesis of A Secondary Aniline from Nitroarene and Aldehyde

**Scheme 1:**
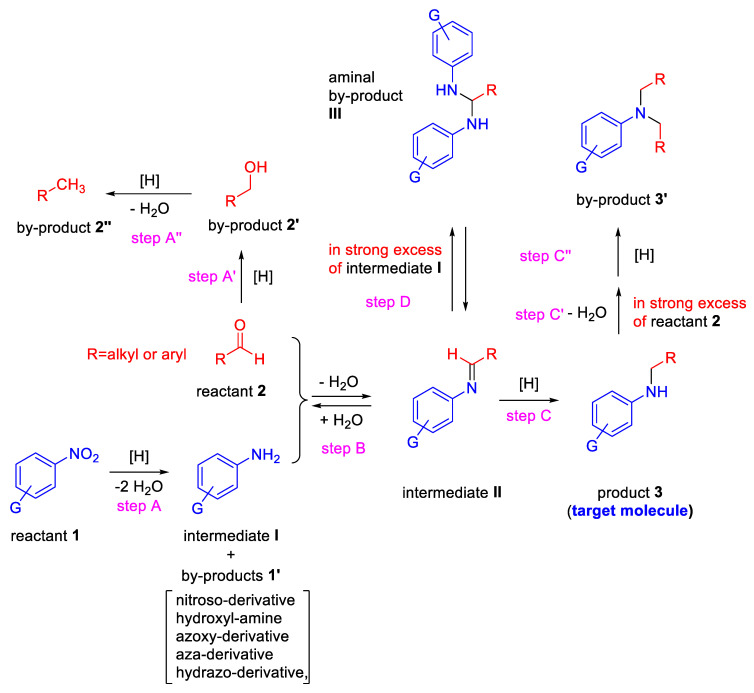
Key steps for the one-pot reductive amination from nitroarenes and aldehydes.

Before reviewing the various types of metal-based heterogeneous catalysts for the title reaction, it is important to highlight the key steps for achieving, in one-pot, a secondary aniline from nitroarene and aldehyde (Scheme 1).

The reaction can formally be regarded as occurring when a nitroarene (reactant **1**, in Scheme 1) is reduced to intermediate **I** (an aniline) which condenses with an aldehyde (reactant **2**) to give an imine (intermediate **II**), followed by reduction of its C═N bond for yielding the secondary aniline (product **3**, the target molecule). In principle, an efficient transition metal catalyst in the presence of an appropriate reducing agent should be able to promote selectively only steps A, B and C. However, several chemo-selective obstacles can arise. Already in step A, together with the formation of intermediate **I**, several by-products (compounds **1′** in Scheme 1) could arise from the generally accepted reaction pathways for the reduction of -NO_2_ to -NH_2_ group [25,26,27,28,29]. In addition, reactant **2** can in parallel be reduced to alcohol (by-product **2′**) in the presence of several common reducing agents and/or transition metals [9,10].

Furthermore, by-product **2′** can be reduced to the corresponding methyl derivative **2″** (hydrogenolysis reaction) provided by a source of acidity, generally furnished by the support of the heterogeneous catalyst used [30,31]. Moreover, if other reducible groups are placed in the nitroarene ring, it could even be difficult to catalytically reduce in a selective manner the nitro functionality [32,33]. In step B, the factors influencing the equilibrium between imine **II** and its corresponding precursors **I** and **2** (solvent, concentration, pH, temperature, electronic and steric effects) should be carefully considered too [9,10,34]. Eventually, in strong excess of reactant **2** and at high temperature, the target molecule **3** (and intermediate **II**) can potentially undergo further RA to yield by-product **3′**, an unwanted tertiary amine [35]. Even when the primary aniline (intermediate **I**) is in strong excess, the intermediate **II** could give an aminal by-product (**III**, step D, Scheme 1), especially in the presence of some protic acids, often used to shift the step B equilibrium towards the imine formation [36].

The rates of the steps reported in Scheme 1 determine the procedure (direct or stepwise) to be followed for a successful one-pot RA promoted by a given catalytic system.

In fact, for the achievement of a DRA starting from nitroarene and aldehyde, the reduction rate of the carbonyl towards alcohol must be considerably slower compared to those of nitroarene and imine intermediate. At this purpose, it is of crucial importance the choice of both metal and appropriate reducing agent, that should catalyze the chemo-selective reduction of the nitro group and imine without touching the aldehyde (or other reducible groups present in reactant **1**). In this context, also the molar ratio of nitroarene and aldehyde plays a key role.

In the case of DRA, the catalytic experiments are generally carried out by using a nitroarene/aldehyde molar ratio other than 1. In some catalytic systems, the nitroarene/aldehyde molar ratio used is less than 1 for neutralizing the effects of the unwanted step A’ on the final yield, while in other catalytic systems it is higher than 1, for increasing the reaction rate of the aniline formation, since its kinetic is dependent by nitroarene concentration.

In the case of stepwise RA, nitroarene and aldehyde are often used in equimolar amount. Sometimes a slight excess of aldehyde is employed to shift the step B equilibrium towards the imine.

### 1.3. Mechanism Pathways of the Nitroarene Reduction (Step A of Scheme 1)

**Scheme 2:**
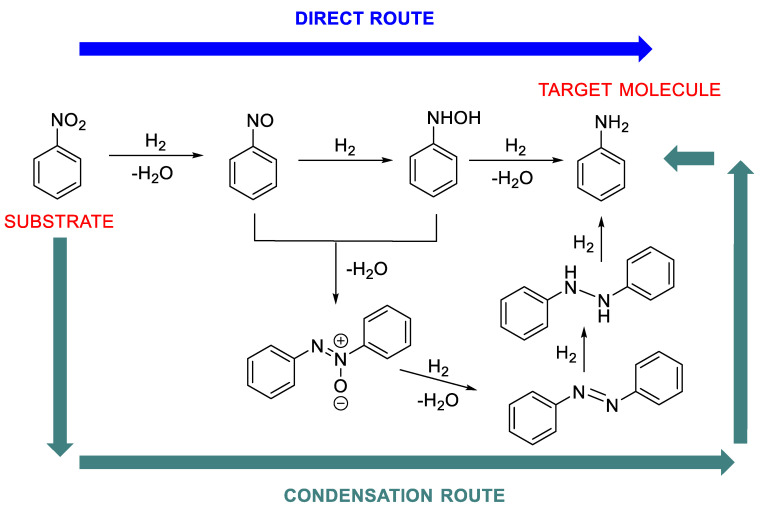
Mechanism pathways for the nitroarene reduction.

Another important aspect that greatly affects the choice of metal and the whole catalytic system to be used in RA is the reaction mechanism of the aniline formation (step A, Scheme 1), which is widely recognized to follow two different (but not mutually exclusive) pathways: the direct and the condensation route (Scheme 2) [37]. The predominance of a pathway on the other one depends on the metal and the reductant used and determines the fate of the overall one-pot RA and the effective type of its procedure, direct or stepwise. For example, it is known that with Pd, Pt or Au based catalysts the direct route (Scheme 2) occurs, while, in the presence of Ni-catalyst, the condensation route (Scheme 2) predominates. For other metals, the followed route depends on the reaction conditions employed. The presence of electron-withdrawing substituents on nitroarene ring has a beneficial effect on nitroarene reduction.

### 1.4. Mechanism Pathways of the Imine Formation (Step B of Scheme 1)

**Scheme 3:**
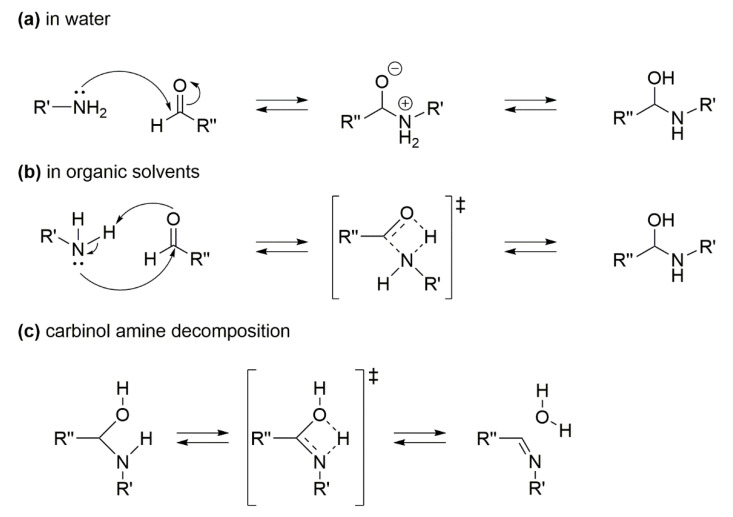
Mechanism pathway for the imine formation: (**a**) carbinol amine formation in water; (**b**) carbinol amine formation in organic solvents; (**c**) carbinol amine decomposition.

The mechanism of the imine formation (step B, Scheme 1) is very important in choosing the catalytic system for a successful RA. The generally accepted pathway for step B consists of two steps [36]: the first one is the formation of carbinol amine via. nucleophilic attack of the amine group to C=O carbon nucleus (Scheme 3a,b), and the second one is the decomposition of the carbinol amine intermediate through elimination of water (Scheme 3c). The first step is affected by the solvent used, as in water solvent it occurs with formation of ionic intermediates (Scheme 3a), while in organic solvents the intermediates are neutral (Scheme 3b). However, the condensation of aldehyde and aniline is an equilibrium reaction, and the formation of the imine is favored by several parameters, such as low pH, high temperature, use of polar solvents. Indeed, the reason why most catalytic systems avoid the use of water as the solvent for the overall one-pot catalytic RA reaction stands in step B equilibrium that would be shifted towards substrates in the presence of water. This drawback should be kept in mind when designing a sustainable RA process, and the present review will emphasize the few catalytic systems that allow the use of water as the solvent.

Notably, contrary to what happens for step A reaction, the presence of electron-donating substituents on nitroarene (transformed into aniline) ring positively affects the step B reaction rate. In addition, the acid features of the support of a heterogeneous metal catalyst (such as a zeolite) could sensibly shift the step B equilibrium towards the imine formation.

### 1.5. Kinetic Considerations on the Imine Hydrogenation (Step C of Scheme 1)

The rate of the imine hydrogenation step (step C, Scheme 1) depends on several factors, such as: the type of metal used, the solvent, the temperature, and the kind of reductant, which can be dihydrogen, a carboxylic acid (mostly formic acid), the CO/H_2_O mixture, sodium borohydride, and others. Substituents in *ortho-*positions on both benzaldehyde and starting nitroarene rings could lower the yield into the final product for steric reasons. In any case, the key step is the metal-hydride formation species. Especially for nanostructured heterogeneous metal-catalysts, the type of support is of crucial importance because it can assist the formation of the metal hydride. An example is the use of doping the insoluble matrix with Brønsted base sites (such as nitrogen centers), which promote the heterolytic splitting of dihydrogen, facilitating the formation of an adjacent metal nanoparticle-hydride. Notably, steps A and C (Scheme 1) are favored by supports with basic features, while step B is facilitated by insoluble matrices with acid properties. Indeed, for the polar nature of the C=N double bond to be hydrogenated, in most cases, the dihydrogen addition is believed to follow the heterolytic pathway, instead of the homolytic one, especially in the presence of polar solvents [38]. In these cases, electron-donating and electron-withdrawing substituents in the starting nitroarene and benzaldehyde rings, respectively, favor the success of step C.

The above considerations point out that the seeming simplicity of the catalytic one-pot secondary amine synthesis starting from nitroarene and benzaldehyde hides a complexity which is the subject of this review, wherein we will try to address strengths and weaknesses of the metal based recyclable heterogeneous catalytic systems described in the last two decades.

## 2. Metal-Catalyzed One-pot Reductive Amination Reaction

In this section the described processes have been subdivided according to the specific metal used as the catalyst, since each metal has peculiar features affecting the mechanism pathway and the type of procedure (direct or stepwise) generally followed. RA reactions catalyzed by platinum group metals are discussed first; then catalytic systems based on Group 11 metals are addressed; and finally, systems comprising transition metals of the first row of the VIII group, such as Fe, Co and Ni are discussed. An example with MoS_2_ and interesting heterobimetallic systems conclude this section.

### 2.1. Platinum Group Metals: Palladium

Palladium is by far the most versatile and widely used metal among transition metals employed in catalysis. In all cases, for this kind of catalysts the catalytically active species have proven to be Pd nanoparticles (NPs) supported on various insoluble matrices. Generally, the catalysts kept their activity and selectivity over several subsequent runs and the reported studies differ from each other for the employed reductant and/or the dimension distribution of the metal NPs.

As recently highlighted by Sukhorukov [22], the RA of carbonyl derivatives with nitro compounds can viewed as an old reaction because more than 80 years ago Major [39] and Emerson et al. [40,41] reported on the coupling of nitroarenes with aldehydes under catalytic hydrogenation over platinum oxide (PtO_2_: Adams’ catalyst [42]) and Raney Nickel catalyst, respectively. The proposed protocols were generally applicable to both aromatic and aliphatic aldehydes but in most cases low or moderate yields in secondary amines were achieved.

**Scheme 4:**
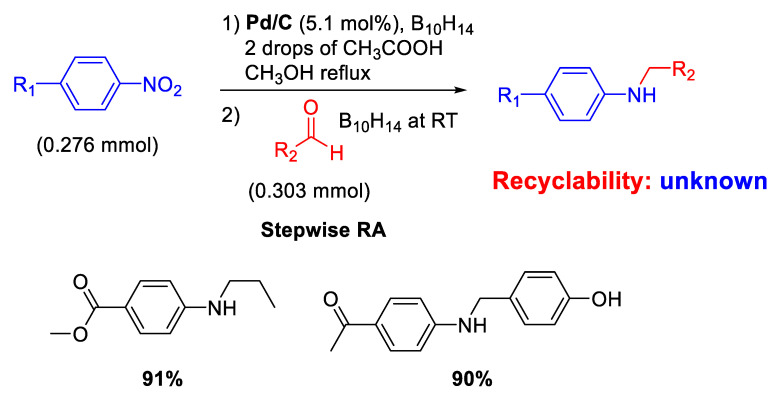
Stepwise RA catalyzed by Pd/C in the presence of decaborane.

The modern revaluation for the employment of heterogeneous catalysis for carrying out RA of aldehydes with nitroarenes started only in the 2000 s with the use of commercial source of palladium catalyst (Pd/C). In fact, Yoon and co-workers reported that Pd/C promoted the conversion of nitroaromatics and aldehydes into *N*-alkylaminoaromatics employing decaborane (B_10_H_14_) as a reducing agent (Scheme 4) [43].

A simple and efficient stepwise RA was achieved throughout reduction of nitroarenes followed by reductive amination. Although the proposed protocol resulted chemoselective and could also be extended to ketones, neither investigations of the scope of reaction nor data on recyclability of catalyst were reported. However, in a follow-up paper [44], the same group was able to extend their protocol under excess of formaldehyde for the synthesis of tertiary amines in high yields.

**Scheme 5:**
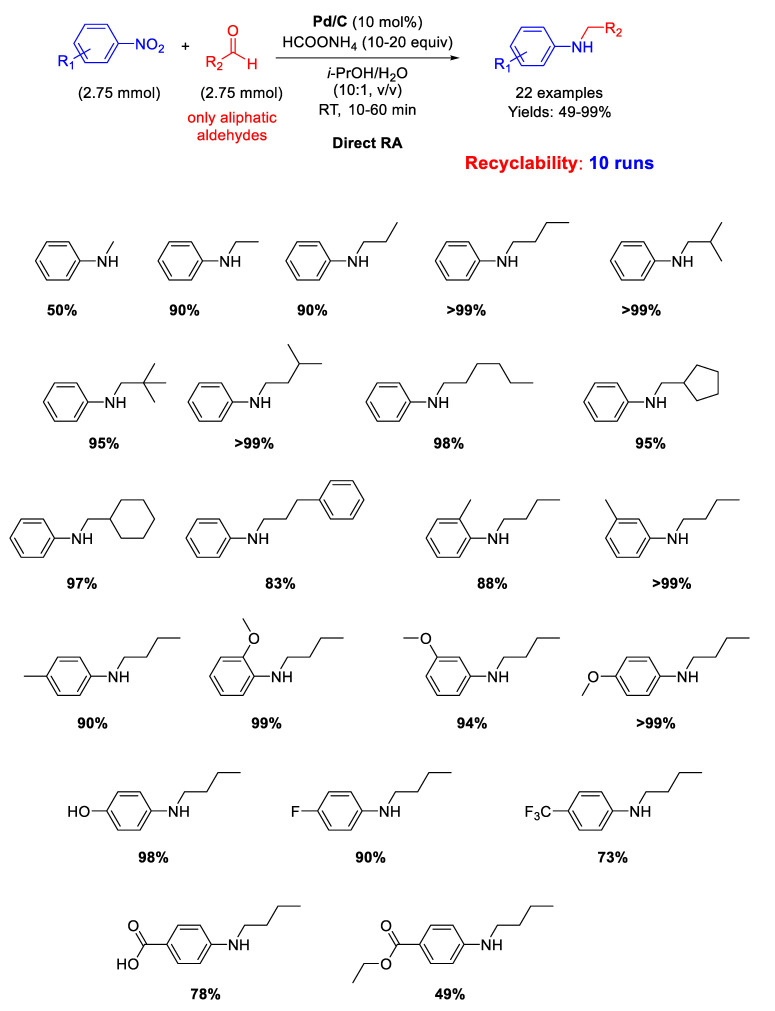
DRA catalyzed by Pd/C in the presence of ammonium formate.

In 2007, Rhee and co-workers proposed another protocol for DRA accelerated by Pd/C [45]. Twenty-two mono-*N*-alkylation of anilines in moderate to excellent yield (49%–99%) were obtained at room temperature by starting from nitroarenes and aliphatic aldehydes in aqueous 2-propanol solvent with ammonium formate (HCOONH_4_), as in situ hydrogen donor (Scheme 5). Moreover, it was demonstrated that Pd/C catalyst could be reused for 10 cycles. The proposed protocol, although facile and environmentally benign, is unfortunately not applicable to aromatic aldehydes.

**Scheme 6:**
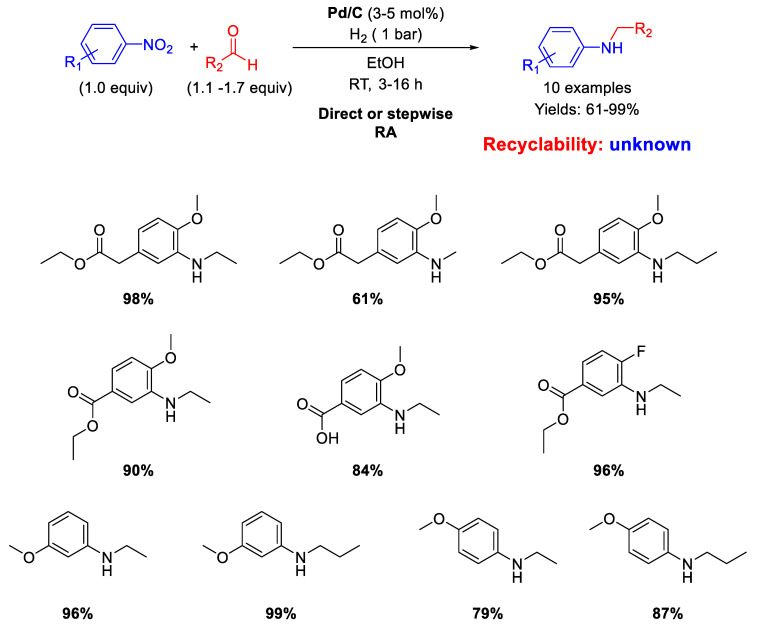
RA catalyzed by Pd/C under H_2_.

Another RA of nitroarenes with aliphatic aldehydes catalyzed by Pd/C which furnished good to excellent isolated yields (61%−99%) was proposed by Sydnes and Isobe in 2008 (Scheme 6) [46].

However, when formaldehyde was used, formation of substantial amounts of the corresponding tertiary amine was observed, but in this case the authors found out that a stepwise RA could be applied with satisfactory yield. No data on the recyclability of the catalyst were given.

**Scheme 7:**
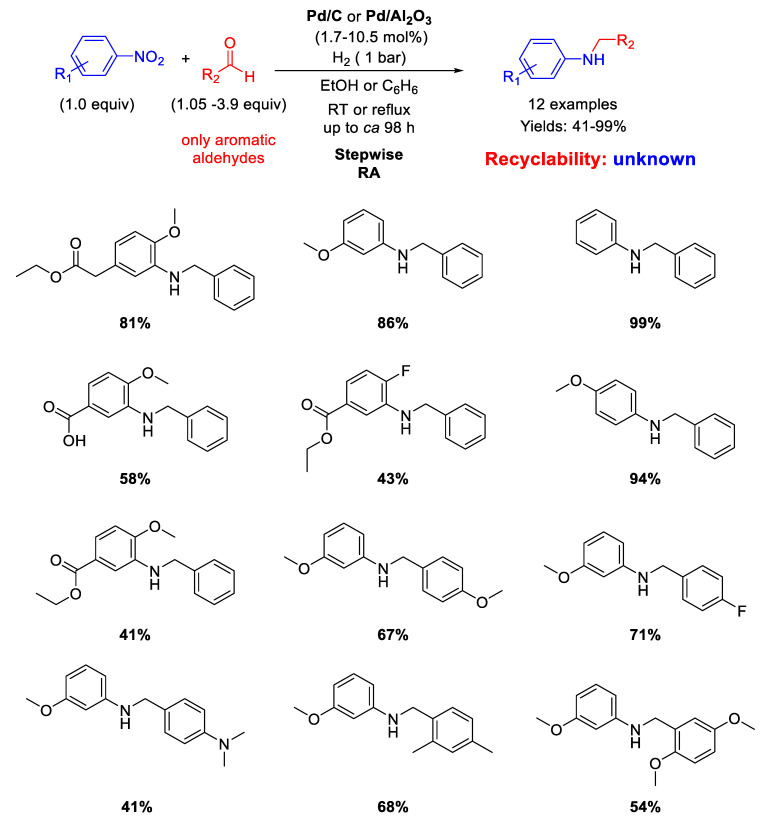
Stepwise RA catalyzed by Pd/C or Pd/Al_2_O_3_ under H_2_.

Sydnes and co-workers, in a follow-up paper [47], extended the scope of their stepwise RA to aromatic aldehydes (Scheme 7). Isopropanol was used as solvent in place of ethanol. However, for the substrates giving a particularly slow imine-forming step, benzene (or benzene/ethanol) under refluxing conditions was employed as solvent for improving the total yields. In these cases, Pd/C was replaced with Pd/Al_2_O_3_ due to the greater stability of the latter catalyst at high temperatures with respect to Pd/C [48]. Sometimes, it was found beneficial to add 0.5 equiv (with respect to nitroarene) of triethylamine before the final imine reduction. Twelve benzylated anilines in yields ranging from 41% to 99% were obtained and some case of low yield was attributed to the substitution patterns associated with the aromatic aldehydes. Once again, no investigations on the recyclability of the catalyst were made.

**Scheme 8:**
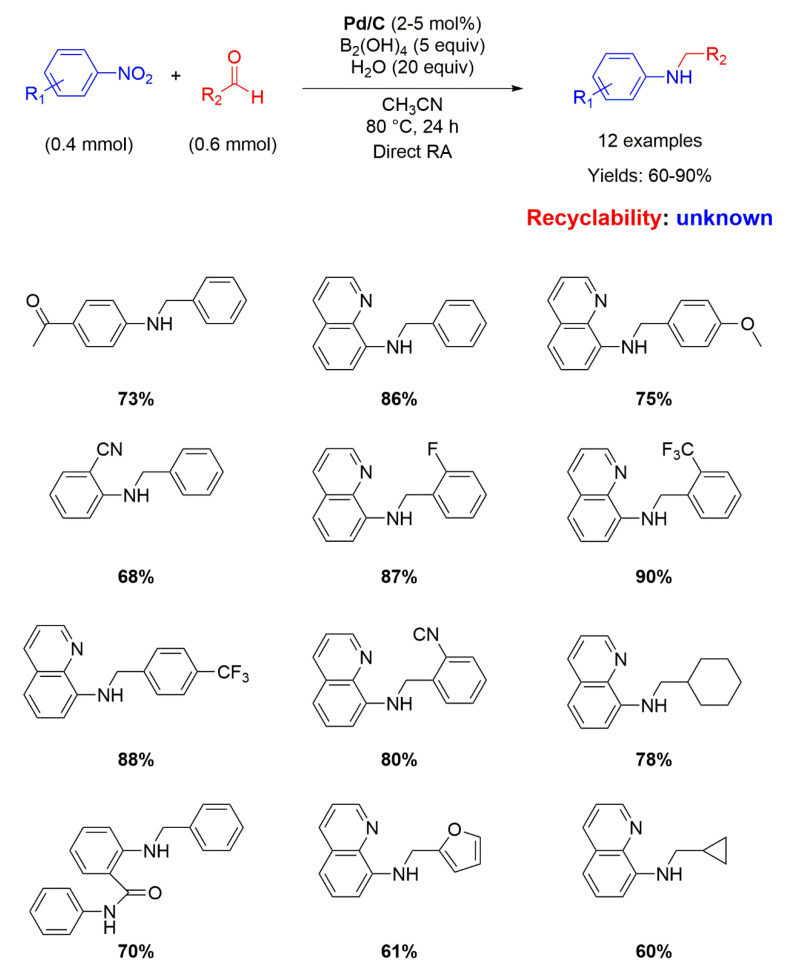
DRA catalyzed by Pd/C with B_2_(OH)_4_ and H_2_O.

Nine years later, Pd/C has been employed again as catalyst for RA, but this time in the direct (not stepwise) fashion. Indeed, Zhou and co-workers used water (20 equiv.) as the hydrogen source mediated by diboronic acid (B_2_(OH)_4_ 5 equiv.) in acetonitrile at 80° for 24 h for reacting various nitroarenes with different aromatic and aliphatic aldehydes, affording yields ranging from 60 to 90% (Scheme 8) [49]. The yields were higher when the substituent in the benzaldehyde ring was electron withdrawing. The role of water as hydrogen donor was verified by using D_2_O as transfer agent. No recyclability studies on the catalytic system have been carried out.

**Scheme 9:**
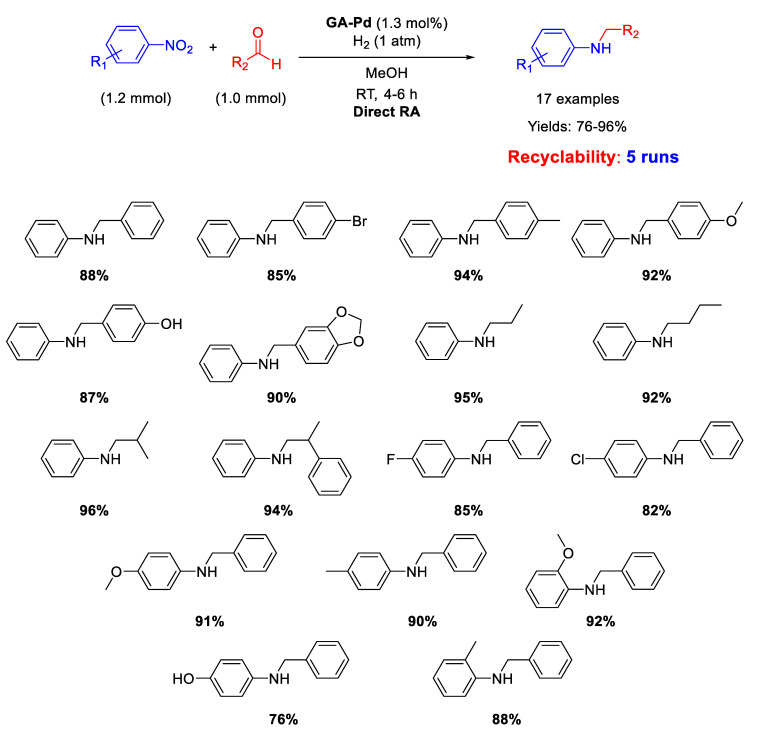
DRA catalyzed by Gum-Acacia supported Pd NPs.

Another important kind of palladium-based heterogeneous catalysts effective for RA is constituted by Pd NPs supported onto organic naturally derived matrices. Pd NPs supported on Gum-Acacia colloids (GA-Pd) have been reported by Sreedhar and co-workers for successful Pd-catalysed DRA applied to both aromatic and aliphatic aldehydes [50]. The supported Pd NPs with a narrow size distribution (ranging from 6 to 12 nm) resulted active and selective towards the formation of secondary anilines for at least five subsequent cycles at room temperature, in methanol solvent, using 1 atm dihydrogen as the reductant (Scheme 9). The yields were quite high (76–96%).

**Scheme 10:**
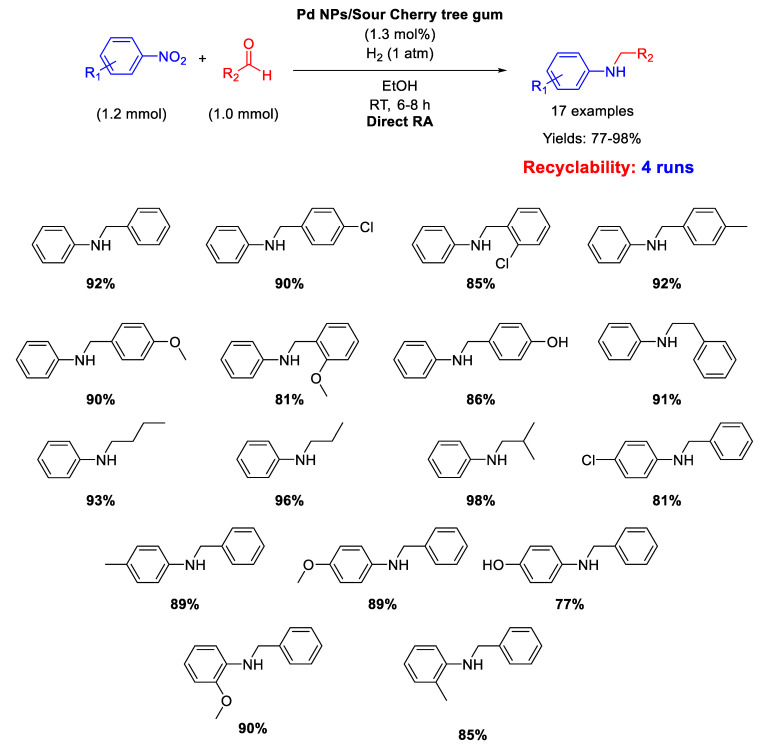
DRA catalyzed by Pd NPs supported onto Sour Cherry tree gum.

A similar catalyst has been prepared by Nasrollahzadeh, that reported on the synthesis of Pd nanoparticles, with size ranging from 2.5 to 15 nm, stabilized on the surface of Sour Cherry tree gum and their use in catalysis for the direct one-pot RA in ethanol at room temperature under 1 atm H_2_ [51]. The catalytic system was active, selective, and recyclable for up to four subsequent runs (Scheme 10).

Pd NPs stabilised by naturally occurring cinchona-based alkaloids, such as cinchonidine, cinchonine, quinine and quinidine, have been reported as catalysts for the one-pot three-step sequential process for the synthesis of *N*-substituted anilines under 3 bar dihydrogen in glycerol at 100 °C [52]. The yields of secondary anilines ranged from 62 to 89%. No recyclability experiments have been carried out on the RA with this catalytic system.

**Scheme 11:**
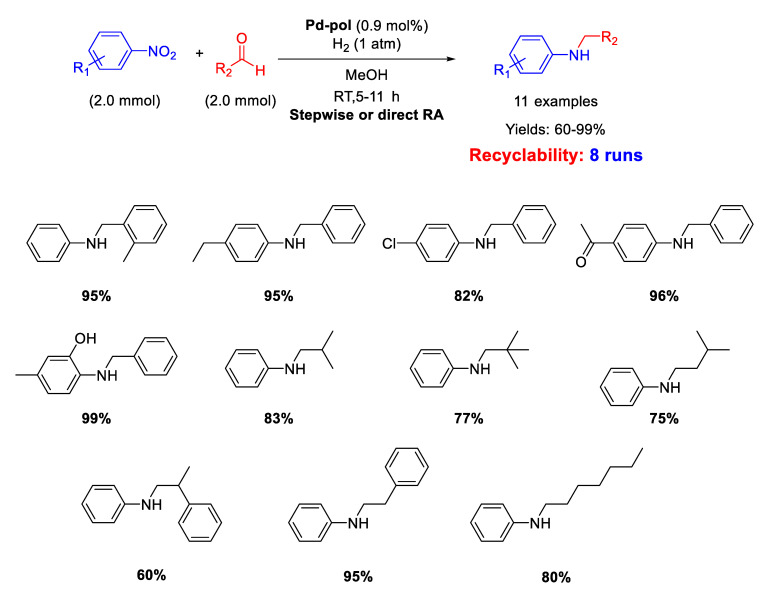
RA catalyzed by Pd-pol.

Dell’Anna and co-workers reported the catalytic activity and recyclability of polymer supported Pd nanoparticles (Pd-pol) obtained by copolymerization of Pd(AAEMA)_2_ [AAEMA^−^ = deprotonated form of 2-(acetoacetoxy) ethyl methacrylate] with methacrylic comonomers, followed by reduction under reaction conditions [53]. Pd-pol also proved to accelerate other kinds of chemical reactions such as reductions, oxidations and esterification. [54,55,56,57,58,59] However, the Pd-pol catalysed reductive amination was successful in one step by using aliphatic aldehydes only. In fact, when benzaldehydes were employed, the one-pot reaction was carried out in three steps to avoid benzyl alcohol formation (Scheme 11). The catalytically active species were in situ formed Pd NPs with a narrow size distribution centred at 5 nm in diameter, whose morphology remained almost unchanged during reaction. The catalyst resulted recyclable for at least eight subsequent runs without loss of activity and selectivity. The catalytic reactions were performed under mild conditions (1 atm H_2_, room temperature, methanol as the solvent) by using equimolar amounts of nitroarene and aldehyde.

**Scheme 12:**
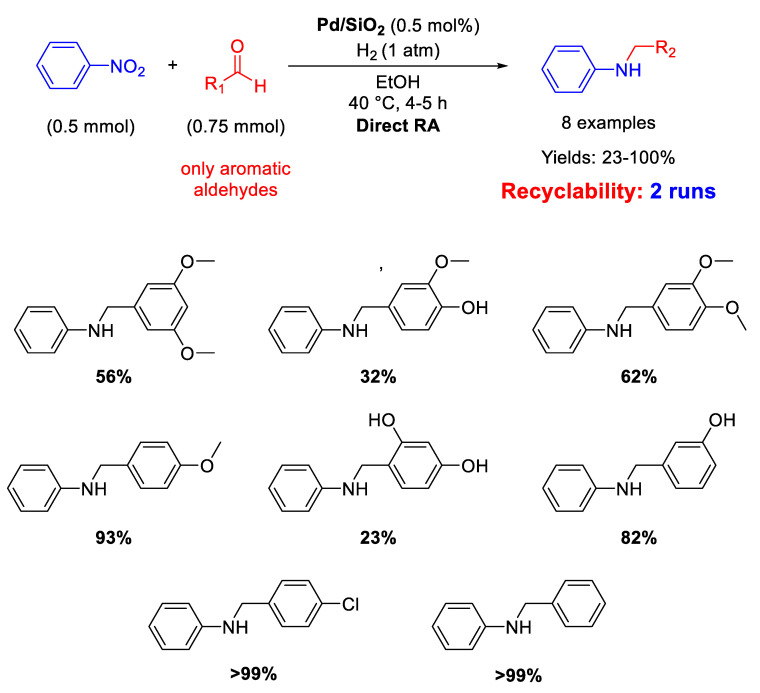
DRA catalyzed by Pd/SiO_2_.

Pd NPs dispersed onto inorganic supports, such as silica and zeolite, have been successfully used as catalysts for DRA. In details, Ma and co-workers reported that SiO_2_ supported Pd nanoparticles with a mean diameter of 5 ± 2 nm were active and selective in the one-pot DRA starting from benzaldehydes and nitroarenes under H_2_, at 40 °C in ethanol (Scheme 12) [60]. The catalytic system gave poor yield (23%) in the desired product, when the benzaldehyde had a substituent in *ortho* position, due to steric hindrance. The catalyst was recyclable for at least two subsequent runs.

**Scheme 13:**
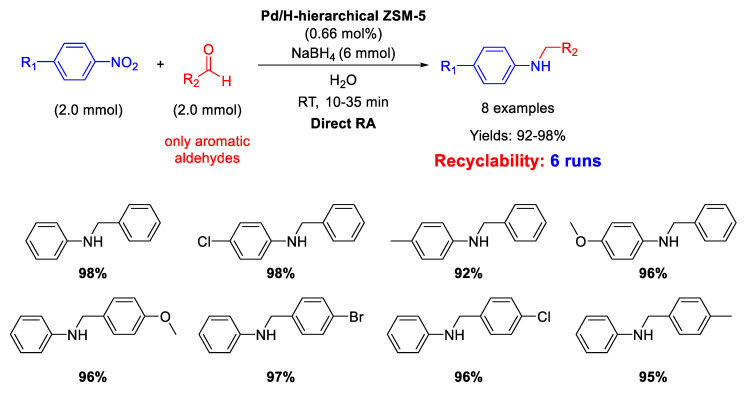
DRA catalyzed by Pd/H- hierarchical ZSM-5.

On the other hand, in 2016, Pd NPs incorporated in ZSM-5 zeolite (ZSM-5 = Zeolite Socony Mobil–5) with a hierarchical structure (Pd/H-hierarchical ZSM-5) have been found effective catalyst for DRA starting from nitroarene compounds, using aldehyde in the presence of NaBH_4_ as a reducing agent (molar ratio of reductant/substrate = 3) and water as the solvent at room temperature (Scheme 13) [61]. Transmission Electron Microscopy (TEM) images showed the formation of an ordered mesostructure in H-hierarchical ZSM-5, supporting Pd NPs with an average diameter of 3–6 nm dispersed in the matrix. The activity of the catalyst was quite high, reaching excellent yields (92%–98%) in short time (10–35 min). Recyclability tests demonstrated that after 6 runs with the same catalyst, the yield into the secondary amine passed from 98% (1^st^ run) to 82% (6^th^ run), and a slight palladium leaching into solution was observed. However, the very interesting aspect of this catalytic system stands in both the use of water as the solvent (which notoriously inhibits the imine formation via condensation of aniline and carbonyl group) and the employing of NaBH_4_ as the reductant, that normally quickly reduces the C=O polar double bond of the aldehyde, even in the absence of metal catalyst, thus subtracting aldehyde for the condensation step reaction. The acid sites and the mesoporous structure of the hierarchical zeolite allowed to overcome all problems associated with the use of water and NaBH_4_. In addition, the authors observed that the reaction rate increased when an electron-withdrawing group was located on the nitroaromatic ring (because the tendency of nitro groups to accept hydride ions increased), or when an electron-donating group was placed on the benzaldehyde ring (because the electron donating group stabilized the protonated carbonyl bond intermediate).

Several research groups synthetized suitable Pd catalysts active in the RDA, by supporting Pd NPs onto Fe_3_O_4_, very often using also SiO_2_ or other inorganic or organic functionalized supports, aiming at taking advantage of the magnetic features of magnetite for recyclability purposes. In all these cases, nitroarene was always used in higher equivalent with respect to aldehyde.

**Scheme 14:**
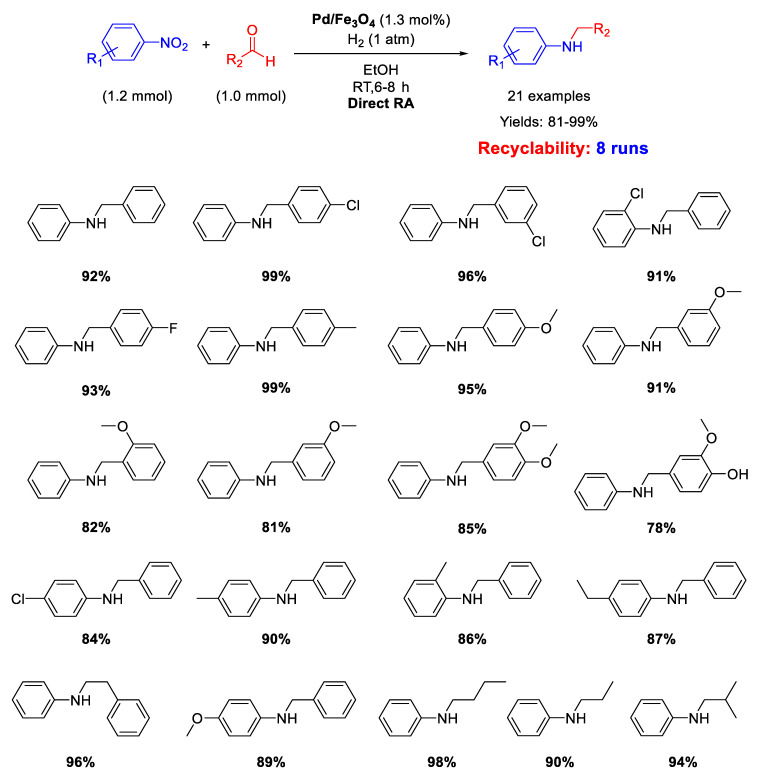
DRA catalyzed by Pd/Fe_3_O_4_.

Ma research group reported on the synthesis of Pd/Fe_3_O_4_ nanocomposites constituted by Pd NPs of 3 nm ca. in size dispersed onto magnetite NPs with an average size of 20 nm [62]. These interesting nano-catalysts were able to catalyze the DRA between benzaldehyde and various nitrobenzenes under hydrogen (1 atm) at room temperature (Scheme 14). Ethanol and methanol were both good solvents, but EtOH was preferred to MeOH from the environmental point of view. The catalyst was easily removed from the reaction mixture thanks to its magnetic features, and then recycled for up eight times without loss of activity and selectivity.

**Scheme 15:**
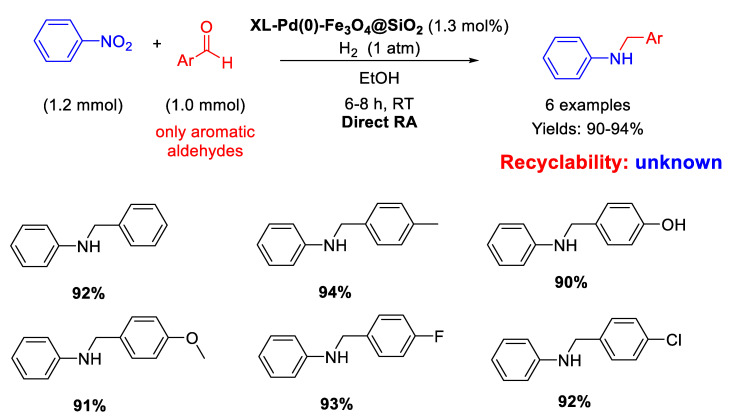
DRA catalyzed by XL-Pd(0)–Fe_3_O_4_@SiO_2_.

In 2014, following their study on Pd nanoparticles supported onto magnetite seeds, Li and Ma and co-workers synthesized with the reverse microemulsion method uncommon nano-catalysts constituted by Fe_3_O_4_@SiO_2_ core-shell nanospheres of 35 nm in size, which were functionalized with ethylenediamine groups in turn reacted with succinyl chloride to support Pd NPs of 5 nm in diameter, homogeneously distributed onto the external matrix surface (XL-Pd(0)–Fe_3_O_4_@SiO_2_) [63]. These nano-catalysts resulted active and selective in the DRA at room temperature in ethanol under 1 atm H_2_ starting from nitrobenzene with various benzaldehydes (Scheme 15). The catalyst was easily recovered at the end of the reaction for further re-uses. The authors noticed that the presence of an electron withdrawing group in *para-* position in the aldehyde ring caused a reduction in the reaction time, while the presence of an electron donating group led to increased reaction times, according to the mechanism proposed in Scheme 3.

**Scheme 16:**
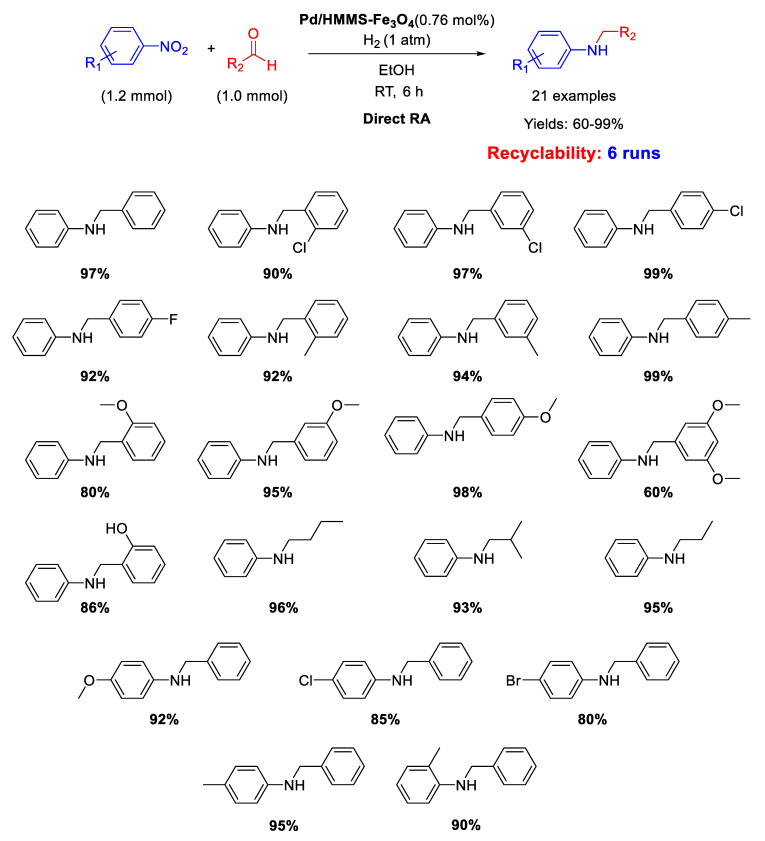
DRA catalyzed by Pd/HMMS-Fe_3_O_4_.

Similar catalytic systems were developed by Li and co-workers for DRA catalyzed by Pd NPs supported on hollow magnetic mesoporous spheres containing Fe_3_O_4_ nanoparticles embedded in a mesoporous silica shell (Pd/HMMS-Fe_3_O_4_) [64]. The Pd NPs with a mean diameter of 7–8 nm resulted homogeneously dispersed onto the surface of the magnetite coated hollow silica sphere. The catalyst could be easily recovered at the end of the reaction, due to its magnetic features, and was reused for six times, without loss of activity. The catalytic tests were carried out in ethanol under dihydrogen atmosphere at room temperature (Scheme 16) and the obtained yields were quite high (60%–99%).

**Scheme 17:**
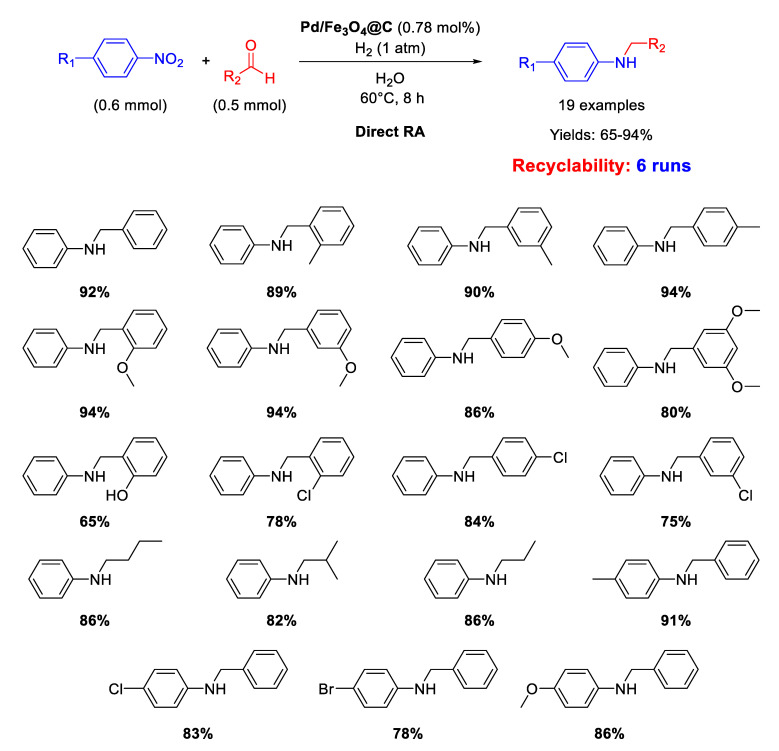
DRA catalyzed by Pd/Fe_3_O_4_@C.

Li’s research group prepared other core–shell Fe_3_O_4_@C nanoparticles (of 160 nm in diameter) having a carbon layer coating of 10 nm in thickness onto which Pd NPs of 3–8 nm in size were homogeneously dispersed (Pd/Fe_3_O_4_@C) [65]. These nano materials represent a rare example of catalysts active for DRA performed in water. Since it was proven the existence of carboxyl groups in the outer carbon layer of these nanocomposites, in our opinion, this occurrence was beneficial for the imine formation step, being the latter favored by acid environment [36], thus allowing the use of water as the solvent. The catalytic experiments were carried out in water at room temperature or at 60 °C under 1 atm dihydrogen. The catalyst could be recycled for up to six subsequent runs (Scheme 17).

**Scheme 18:**
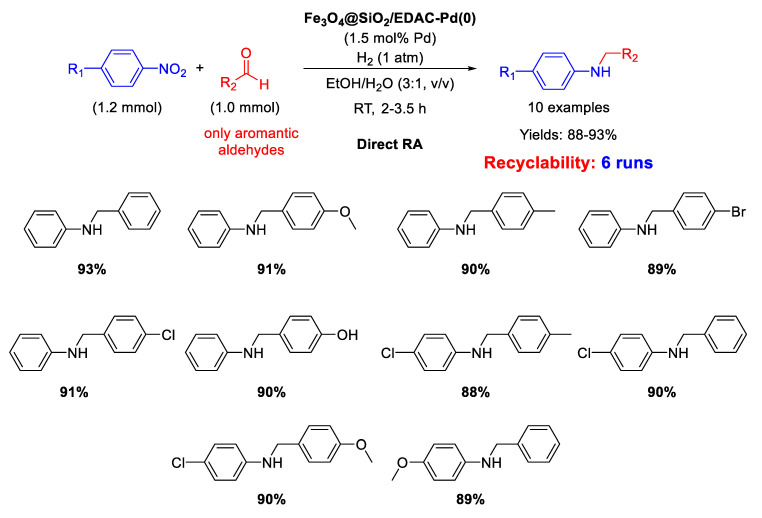
DRA catalyzed by Fe_3_O_4_@SiO_2_/EDAC–Pd(0).

Another example of DRA catalysed by Pd NPs supported onto magnetite based nanocomposites has been reported by Paul’s group, that synthesized in 2016 Pd(0) nanoparticles dispersed onto ethylene diamine functionalized inorganic/organic magnetic composite [66]. In details, Fe_3_O_4_ nanoparticles were prepared with the co-precipitation method, and were coated with silica spheres with the sol-gel process. The final inorganic/organic hybrid magnetite nanoparticles were arranged via formation of covalent bonds between ethylene diamine functionalized cellulose (EDAC) and silica coated Fe_3_O_4_, and then treated with PdCl_2_ and in situ reduced using NaBH_4_ to get Fe_3_O_4_@SiO_2_/EDAC–Pd(0). TEM analyses showed that Pd NPs diameters ranged from 10 to 15 nm and the nano catalysts resulted active, selective, and recyclable for at least six runs in the DRA under 1 atm H_2_, using ethanol/water (3/1, *v*/*v*) as the solvent (Scheme 18). This is another rare example of metal catalysed DRA performed in aqueous medium.

**Scheme 19:**
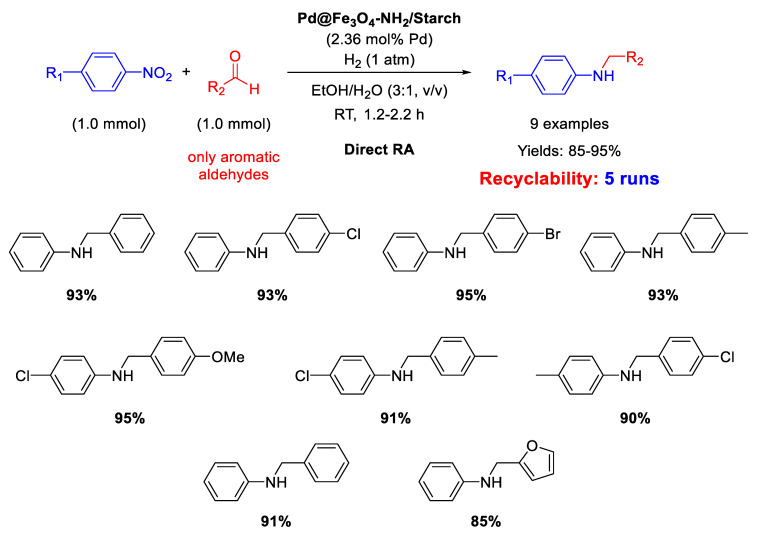
DRA catalyzed by Pd@Fe_3_O_4_-NH_2_/Starch.

In 2017, Paul’s group synthesized another magnetite-based core-shell nanocomposite, useful for catalyzing DRA reaction, namely Pd@Fe_3_O_4_-NH_2_/Starch, which was slightly different from the previously discussed one, because starch was used in the place of cellulose and the coating of silica layer was avoided [67]. Pd NPs deposited onto the amine functionalized starch surface had a mean dimension centered at 8.5 nm in diameter. As for Fe_3_O_4_@SiO_2_/EDAC–Pd(0), Pd@Fe_3_O_4_-NH_2_/Starch was active for the DRA performed in ethanol/water (3/1, *v*/*v*), obtaining good to excellent yield in the desired product (Scheme 19). Recyclability test demonstrated that the catalyst recovered at the end of reaction with an external magnet could be re-used for at least five runs without losing its activity and selectivity.

**Scheme 20:**
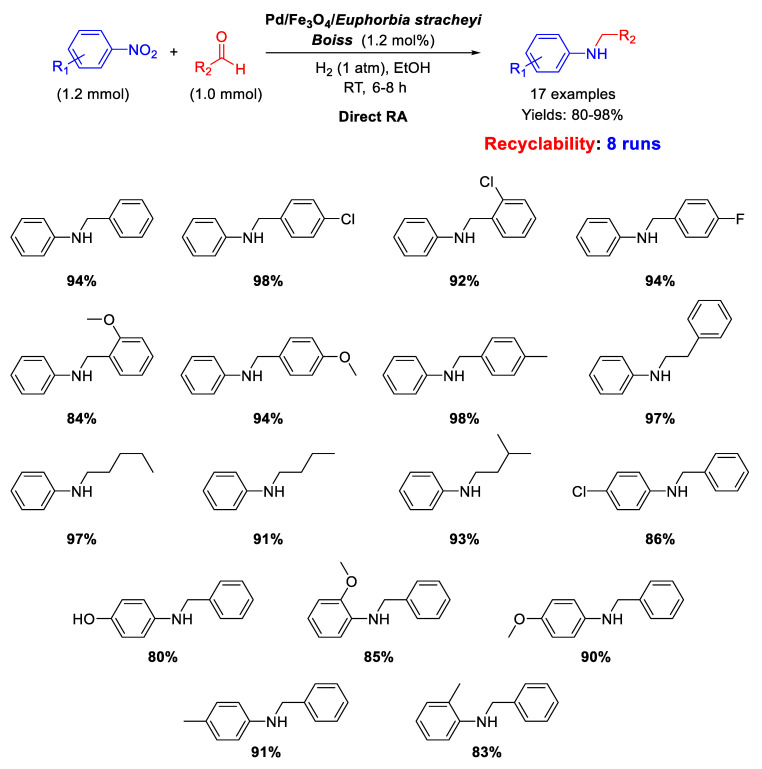
DRA catalyzed by Pd/Fe_3_O_4_/*Euphorbia stracheyi Boiss* root extract.

In the framework of Pd/Fe_3_O_4_ nanocomposites as efficient catalysts for DRA reaction, Nasrollahzadeh’s group reported on the synthesis, characterization, and catalytic activity of palladium and magnetite NPs supported on *Euphorbia stracheyi Boiss* root extract [68]. FESEM micrographs of the catalyst showed that Fe_3_O_4_ and Pd NPs were homogeneously distributed, and TEM images revealed that the obtained nanoparticles had spherical shape with an average diameter of 10 nm. The catalytic system was tested in the DRA of various nitroarenes with benzaldehyde and in the reaction between nitrobenzene with differently substituted benzaldehydes, in ethanol, at room temperature, under 1 atm H_2_, affording good (80%) to excellent (98%) yields in the target product (Scheme 20). Recyclability experiments demonstrated that the catalyst could be easily recovered at the end of reaction thanks to its magnetic properties and could be re-used for at least 8 runs without loss of activity.

**Scheme 21:**
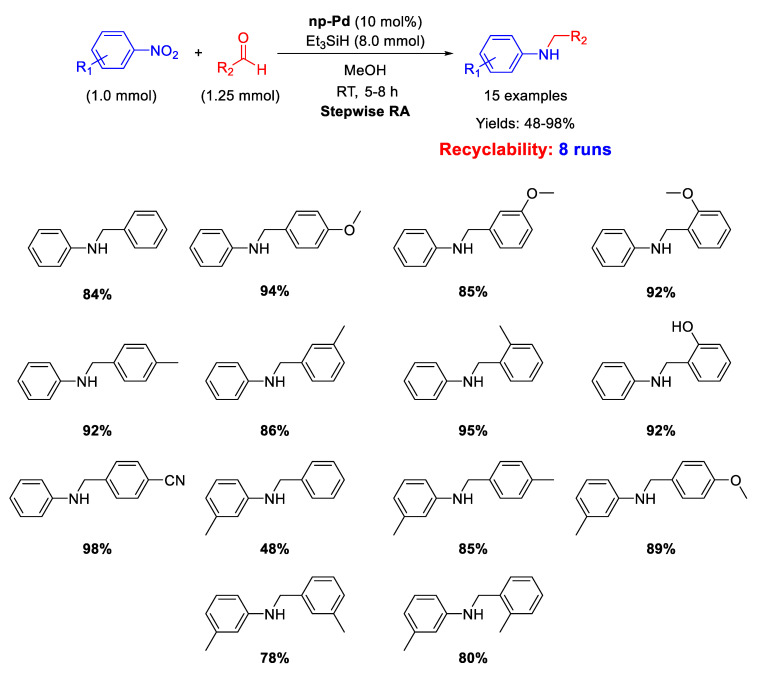
Stepwise RA catalyzed by np-Pd.

Very recently, in 2020 Cao et al. described the catalytic activity of nanoporous palladium (np-Pd) in the stepwise RA [69]. This nanoporous palladium was obtained by dealloying an aluminum-palladium alloy by treatment with a concentrated solution of sodium hydroxide, that caused the leaching of aluminum from the alloy. The catalytic system was constituted by np-Pd (pore size of 5 nm), using triethyl silane (Et_3_SiH) as the hydrogen donor in methanol. The overall reductive amination reaction occurred in one-pot three-step way (Scheme 21). In the first step, the nitroarene was reduced to aniline with the addition of Et_3_SiH; in the second step, benzaldehyde was added to the reaction mixture for the formation of the imine; in the last step, fresh Et_3_SiH was added for the reduction of imine to amine. The catalytic system resulted recyclable for at least 8 runs without losing activity and selectivity. The amount of catalyst employed was, however, unusually high (10 mol% of Pd).

### 2.2. Platinum Group Metals: Platinum

**Scheme 22:**
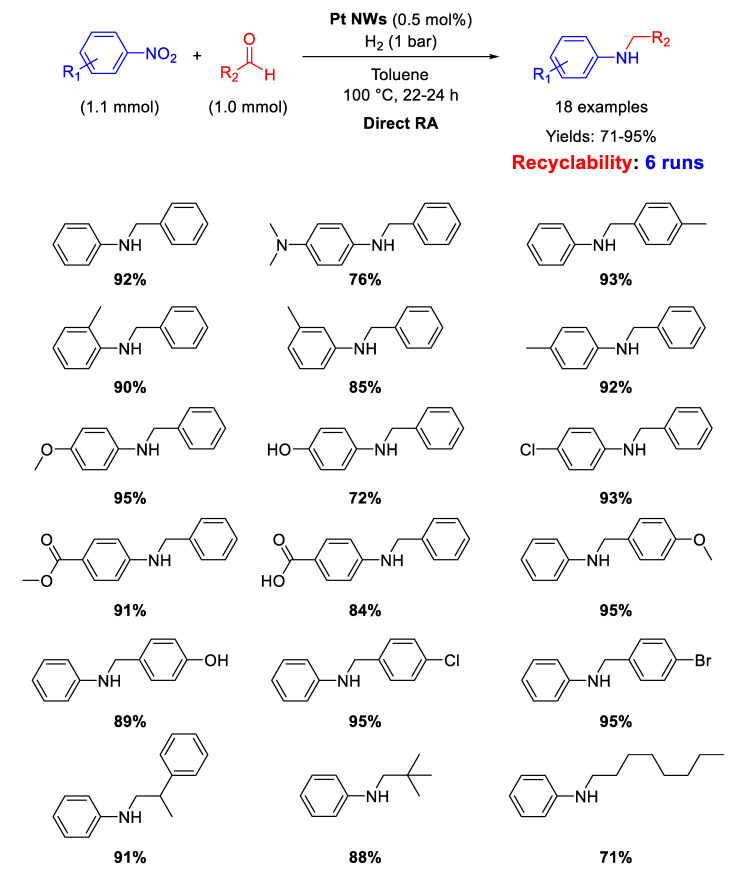
DRA catalyzed by Pt NWs.

Few examples have been reported in recent years in the field of RA catalyzed by platinum-based catalysts. Among them, Lu and Gu and co-workers [70] proposed the use of ultrathin Pt nanowires (Pt NWs) as catalyst for DRA starting from various nitroarenes with different benzaldehydes under 1 atm H_2_, in toluene, at T = 100 °C. The yields in the desired products were quite high, reaching the value of 95% in some cases (Scheme 22). Recyclability tests demonstrated that the catalyst could be recycled for 6 runs, without losing activity and selectivity. TEM analyses showed that Pt NWs were several hundred nm long and had an average diameter of 2 nm. On the base of GC analyses and DFT calculations, the authors proposed an unusual mechanism pathway for the DRA with their catalytic system. Since Pt catalysts (like Pd catalysts) reduce nitroarenes following the direct route reported in Scheme 2, the authors claimed that, in the presence of Pt NWs under H_2_, the hydroxylamine intermediate (see Scheme 2) directly reacts with the carbonyl group of the aldehyde before converting into aniline. In this way, the *N*-substituted secondary aniline would be obtained without passing throughout the formation of imine.

Shreedar and coworker [71], following their study on precious metal catalyzed RA, employed PtO_2_ as pre-catalyst for the stepwise RA starting from various nitrobenzenes, that were first converted into anilines and then condensed with benzaldehydes to give imines. At the end, the imines were hydrogenated to obtain the target product in one-pot three-step way. The overall reaction occurred in methanol at room temperature, by putting the system under 1 atm of H_2_ only in the first and in the third step. The catalytically active species were Pt particles suspended in the reaction medium, obtained by preliminary H_2_ reduction of PtO_2_ suspended in methanol.

**Scheme 23:**
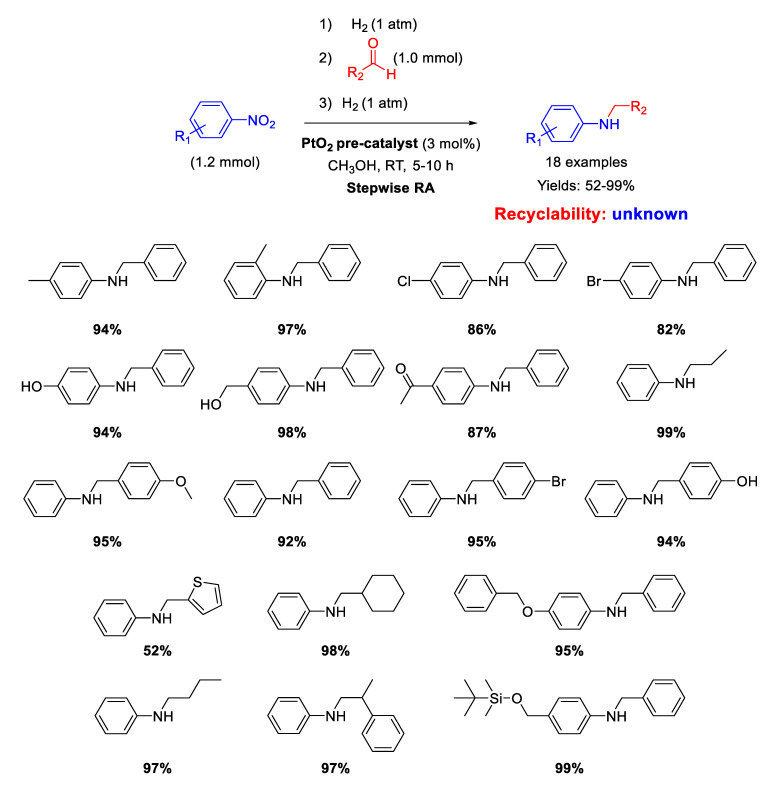
Stepwise RA in the presence of PtO_2_ as pre-catalyst.

No recyclability tests have been reported. Yields ranged from 82 to 99%, being lower (52%) only by using tiophene-2-carboxaldehyde in the RA with nitrobenzene, because sulfur derivatives are known to poison noble metal catalysts (Scheme 23).

**Scheme 24:**
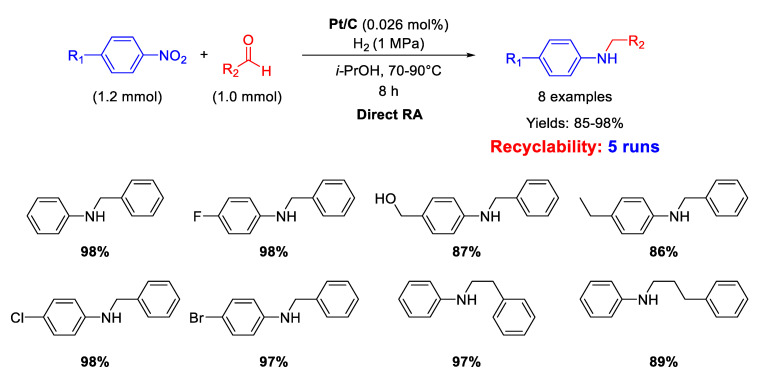
DRA catalyzed by Pt/C.

In 2020 Zhang and coworkers described the synthesis of Pt nanoparticles with average size of 2.9 ± 0.6 nm loaded onto commercial carbon black to obtain Pt/C with 0.5%_w_ of Pt, able to catalyze DRA [72]. The reaction conditions used for catalyzing DRA were quite harsh for a noble metal-based system: T = 70–90 °C, under 1 MPa of H_2_ in isopropanol as the solvent, t = 8 h. Conversely, the metal loading was very low (0.026 mol% of Pt) (Scheme 24). Yields were excellent (85%–98%). Recyclability studies revealed a slight decrease of activity after five subsequent runs with the same catalyst.

### 2.3. Platinum Group Metals: Iridium

**Scheme 25:**
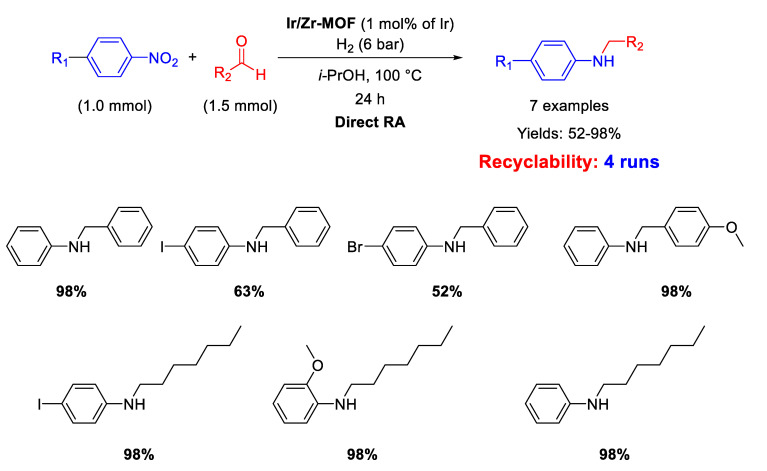
DRA catalysed by Ir/Zr-MOF.

An Ir-based catalyst employed in DRA has been reported by Corma’s group [73]. The catalyst was a multisite metal organic framework (MOF) material, prepared starting from amine-functionalized Zr-based MOFs, loaded with an Ir complex. This new Zr/Ir-MOF nano-compound acted as a hybrid catalyst that combined the properties of soluble organometallic complexes with those of the MOF heterogeneous support. In fact, it resulted active, selective, and recyclable for at least four runs in the DRA of benzaldehyde with nitrobenzene under 6 bar H_2_ at 100 °C in 24 h (Scheme 25). Hot filtration tests demonstrated that the catalytic cycle occurred in the heterogeneous phase, while TEM analyses revealed that no metal nanoparticles formed during the reaction.

**Scheme 26:**
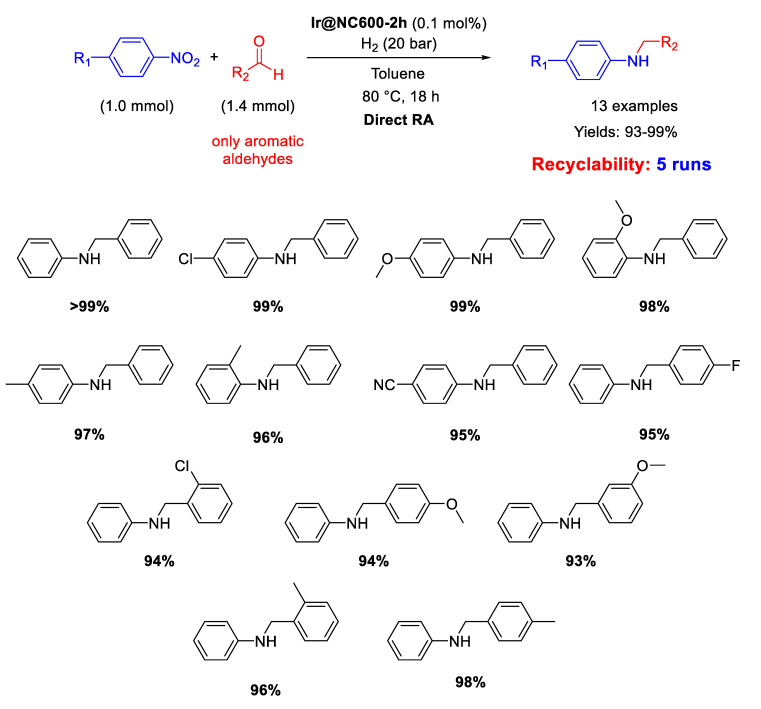
DRA catalysed by Ir@NC600-2h.

In 2017, Huang and co-workers prepared an Ir catalyst (Ir@NC600-2h) by pyrolysis under nitrogen at 600 °C for 2 h of the iridium pre-catalyst, obtained by reaction of the ionic liquid 1-methyl-3-cyanomethylimidazolium chloride with IrCl_3_ in the presence of activate carbon powder, to form Ir NPs of 3–5 nm in size, dispersed in the resulting N-doped carbon material [74]. The catalyst was employed in the DRA of aldehydes with nitroaromatics in toluene at 80 °C under 20 bar H_2_. The obtained yields were excellent (93%–99%) (Scheme 26). Recyclability tests were performed starting from benzaldehyde and aniline (instead of nitrobenzene) and the recovered catalyst was reused for five subsequent runs with a slight decrease in its activity.

### 2.4. Platinum Group Metals: Ruthenium

**Scheme 27:**
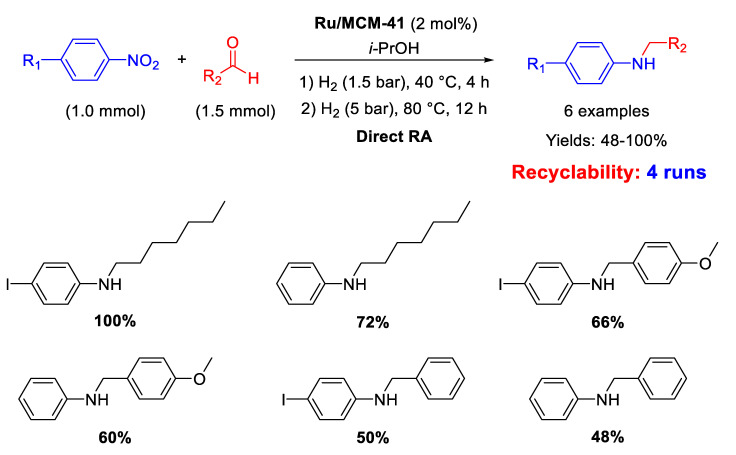
DRA catalysed by Ru/MCM-41.

There is just one recent example of Ru catalyst employed for RA, reported in 2012 by Sánchez and co-workers, that synthesized and immobilized on MCM-41 a Ru(II) hydride complex containing a pincer-type pyridine-functionalized *N*-heterocyclic carbene ligand. This catalyst was used to selectively catalyse the formation of *N*-substituted amines from nitroaromatics and carbonyl compounds [75]. The reactions were carried out in isopropanol in this way: first the nitroarene/carbonyl compound mixture was kept at 40 °C under 1.5 bar H_2_ for 4 h, causing nitroarene reduction and subsequent imine formation, and then the system was heated to 80 °C under 5 bar H_2_ for 12 h for obtaining good yields in the target molecule (Scheme 27). Ethanol could not be used as it resulted in the formation of *N*-ethyl anilines. The acidity of the MCM-41 matrix promoted the formation of the imine intermediate and, for this reason, the supported Ru catalyst resulted more active than its soluble analogue. Recyclability tests showed that the activity of the recovered heterogeneous catalyst did not decrease after 4 cycles.

### 2.5. Platinum Group Metals: Rhodium

**Scheme 28:**
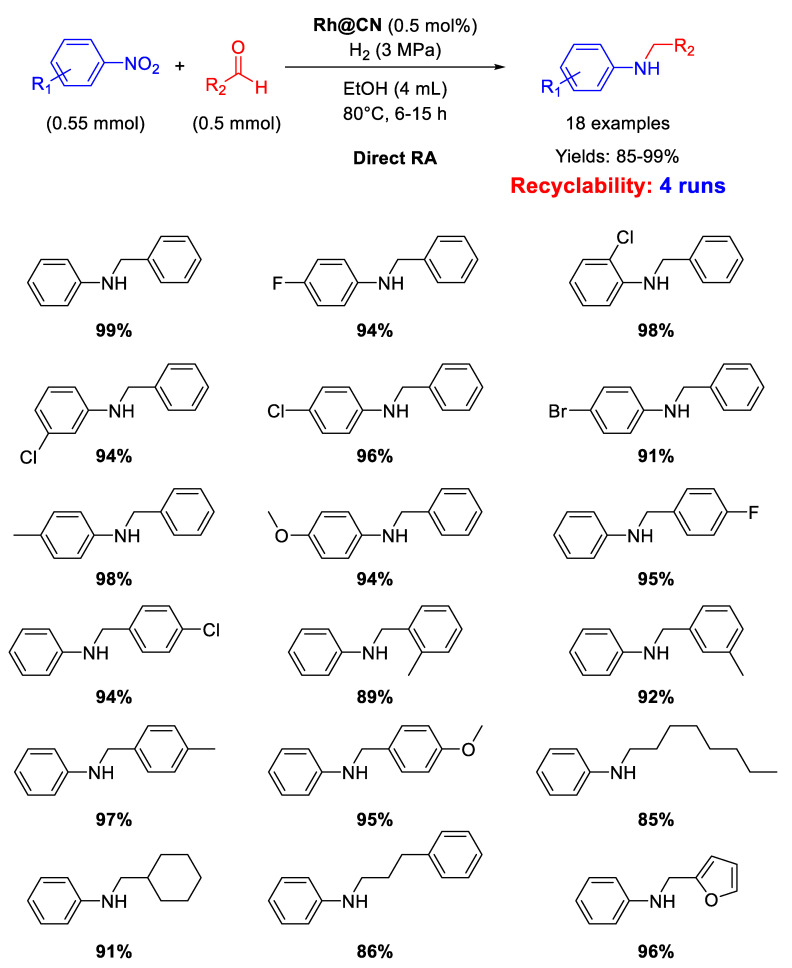
DRA catalysed by Rh@CN.

There is just one example of Rh-based heterogeneous catalysts for RA in recent years. In 2015 Huang and co-workers reported on the synthesis of a Rh pre-catalyst by reaction between RhCl_3_ and 1,10-phenantroline in the presence of activate carbon, following a procedure introduced by Beller’s group for the synthesis of cobalt [76] and iron catalysts [77]. The obtained black solid was calcined at 800 °C for 2 h under N_2_ to get the final catalyst Rh@CN as black powders formed by N-doped carbon supporting Rh nanoparticles of 6 nm in size [78]. Rh@CN was able to catalyze the DRA of differently substituted benzaldehydes with various nitrobenzenes under 3 MPa of H_2_, at 80 °C, in ethanol for 6–15 h (Scheme 28). The catalyst was re-used for four subsequent runs without loss of activity. TEM analyses showed that Rh NPs slightly increased in size (8 nm) after recycles.

### 2.6. Other Noble Metals (Group 11): Gold

**Scheme 29:**
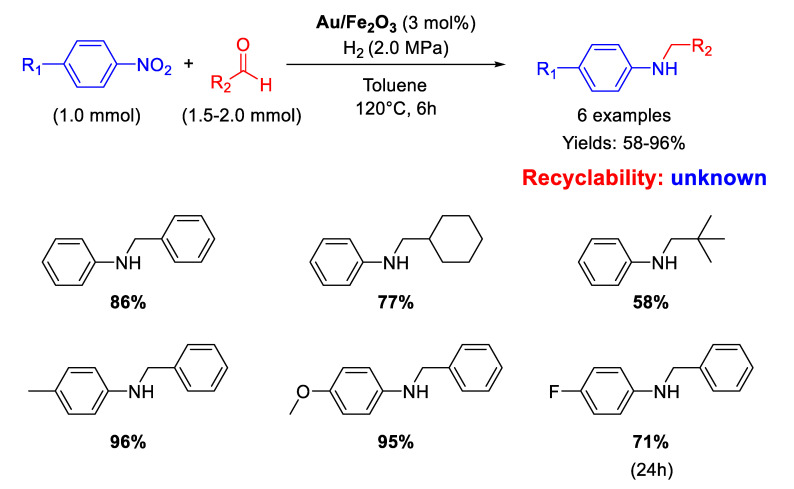
DRA catalysed by Au/Fe_2_O_3_.

Between 2007 and 2009, Corma’s group was the first to understand the potential of gold nanoparticles for promoting several cascade-type reactions for the synthesis of different nitrogen-based compounds [79,80] but they reported only one case of DRA starting from nitroarenes and aldehydes catalyzed by TiO_2_ supported Au NPs. In this framework, in 2009 Tokunaga and co-workers [81] employed Au NPs (2−3 nm in diameter) supported on Fe_2_O_3_ (Au/Fe_2_O_3_) under low hydrogen pressure for one-pot synthesis of aniline derivatives from nitroarenes and aliphatic or aromatic aldehydes in yields from 58 to 96% (Scheme 29). Aliphatic aldehydes gave lower yields while nitroarenes with electron-withdrawing substituents needed longer reaction times. No attempts of recyclability were carried out with the catalyst because Au/Fe_2_O_3_ after the catalytic reaction showed a slightly increase of gold particle size as revealed by TEM investigations.

**Scheme 30:**
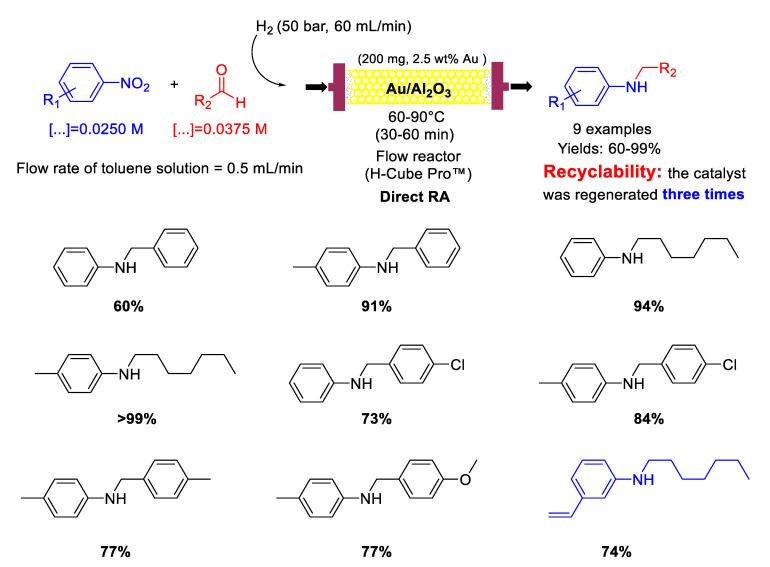
DRA catalysed by Au/Al_2_O_3_ in flow reactor.

In 2015, Nuzhdin and co-workers [82] published their first paper reporting on one-pot reductive amination of aromatic and aliphatic aldehydes with nitroarenes, promoted by Au/Al_2_O_3_ catalyst with a mean Au particle diameter of 3.4 nm in a continuous flow packed bed reactor using high pressure of molecular hydrogen as a reducing agent (Scheme 30). Nine secondary aromatic amines were achieved in good to excellent yields and the catalyst showed remarkably high chemoselectivity towards the synthesis of *N*-heptyl-3-vinylaniline starting from 3-nitrostyrene and *n*-heptaldehyde. In the case of *N*-heptylaniline, the catalyst was regenerated three times by washing every 135 min the catalyst cartridge with a flow of pure methanol (1 mL/min) for 30 min. Between any cycle (reaction and regeneration) only a slight decay in the yield of *N*-heptylaniline was observed without leaching of Au from the Au/Al_2_O_3_ or increasing in gold particle size, but carbon deposition occurred at some extent, as ascertained by differential thermal analysis.

**Scheme 31:**
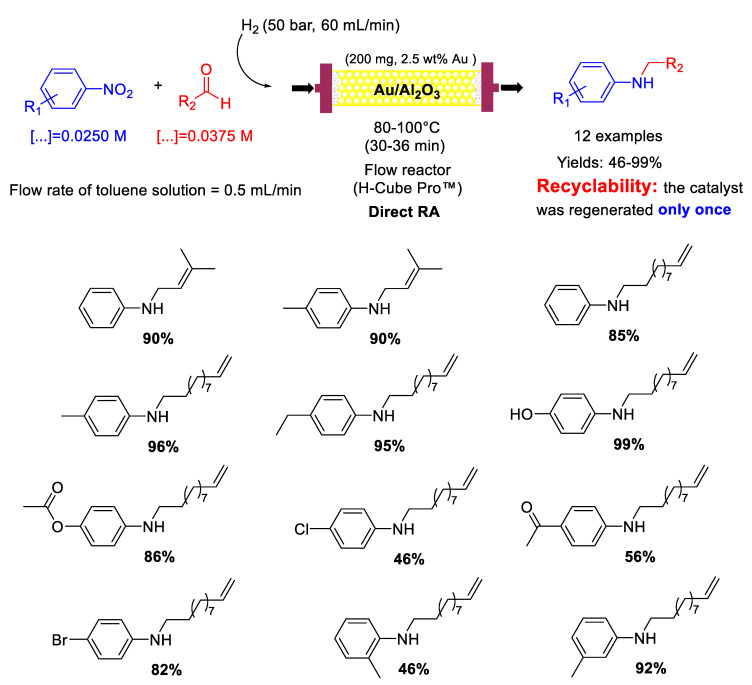
DRA catalyzed by Au/Al_2_O_3_ in flow reactor using unsaturated aliphatic aldehydes.

The high chemoselectivity shown by Au/Al_2_O_3_ paved the way to a follow-up article [83] in which the same group reported that Au/Al_2_O_3_ could also catalyze in a continuous-flow the DRA of aliphatic unsaturated aldehydes with nitroarenes (Scheme 31) obtaining unsaturated secondary amines in moderate to excellent yields (46−99%). Also in this case, the authors were able to regenerate the catalyst by washing it every 150 min with a flow of pure toluene (0.5 mL/min) for 30 min and again it was ascertained by thermal analysis that the formation of carbon-based deposits was the main reason for the slight catalyst deactivation.

**Scheme 32:**
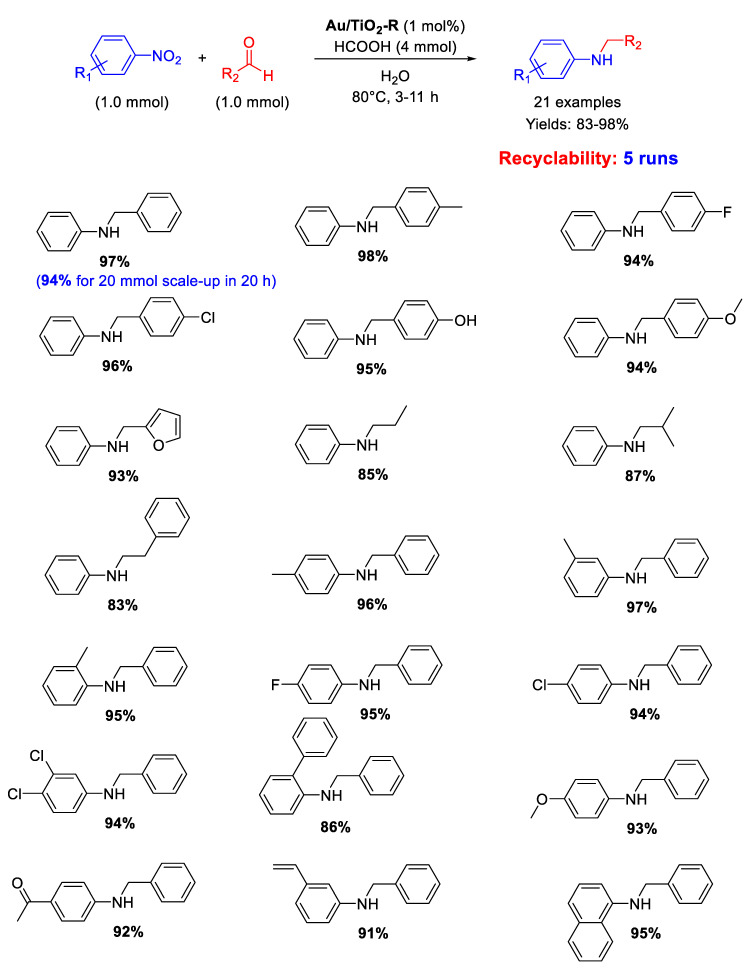
DRA catalyzed by Au/TiO2-R.

Cao and co-workers showed that Au/TiO_2_-R, constituted by Au NPs with an average particle size of 2.2 nm supported on titania in the form of rutile (TiO_2_-R), catalyzed under very mild reaction conditions the chemoselective transfer hydrogenative DRA of aldehydes with nitroarenes in neat water as solvent employing formic acid (FA) as hydrogen donor (Scheme 32) [84]. A broad scope reaction was demonstrated with twenty-one secondary aromatic amines being obtained in good to excellent yields (83–98%) minimizing *N*-formyl derivatives and other unwanted by-products that usually form in appreciable levels with FA-based protocols. Moreover, raising the reaction time up to 20 h, a gram-scale reaction (up to 20 mmol scale-up) was accomplished for the synthesis of *N*-benzylaniline in 94% yield. Au/TiO_2_-R was reused five times without loss of activity and metal leaching. In addition, investigations by TEM, X-ray Photoelectron Spectroscopy (XPS) and X-Ray Diffraction (XRD) revealed almost no change in the dispersion of the Au NPs or metallic state of Au before and after each reuse.

**Scheme 33:**
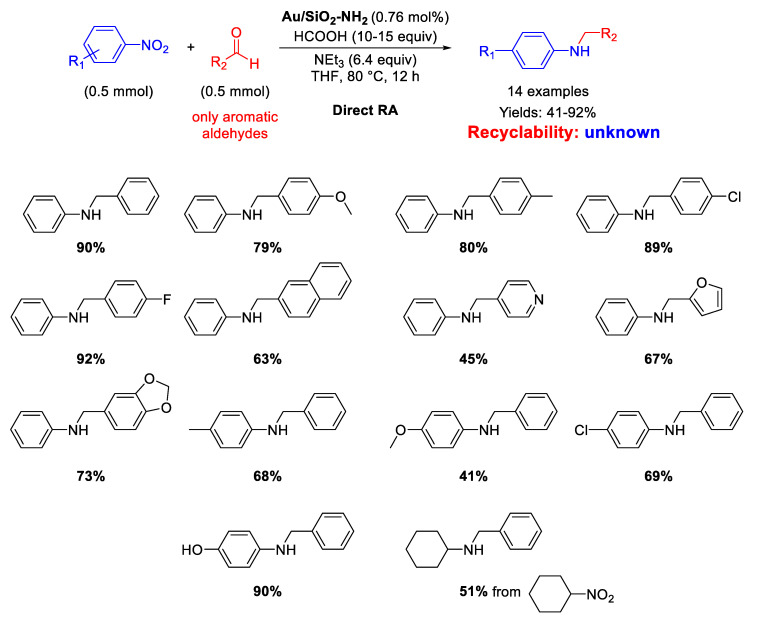
DRA catalyzed by Au/SiO_2_-NH_2_.

In 2020, Rossi and co-workers immobilized pre-synthesized Au NPs by sol-immobilization method on Stöber silica (microspheres of ca 400 nm) obtaining the Au/SiO_2_-NH_2_ catalyst [85], which was employed for both reductions and reductive aminations using formic acid as hydrogen source (Scheme 33). The active species were supported Au NPs with an average particle size of 2.2 nm that catalyzed the formation of fourteen secondary amines in moderate to very good yield (41%–92%). To achieve high reaction rates, it was necessary to employ an appropriate Brønsted base (triethylamine), which acted cooperatively with the gold surface for the activation of formic acid as reducing agent. Although the Au/SiO_2_-NH_2_ catalyst could be reused up to five times without any significant loss of activity and almost no agglomeration of the Au NPs in selective hydrogenation of alkynes, no details on the catalyst recyclability was furnished.

### 2.7. Other Noble Metals (Group 11): Silver

**Scheme 34:**
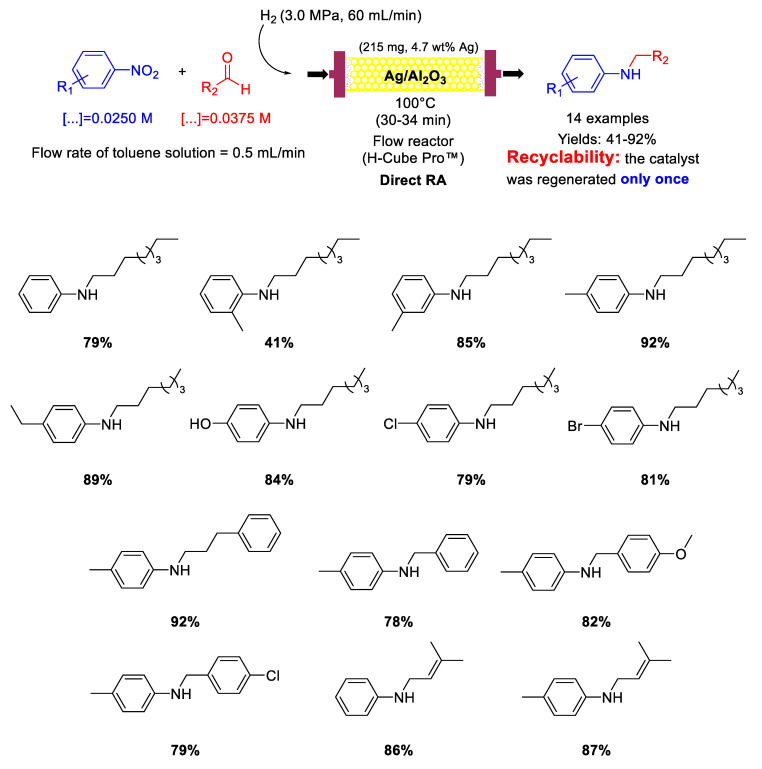
DRA catalyzed by **Ag/γ-Al_2_O_3_** under flow conditions.

The only example of a silver-based catalyst reported in recent years for catalysing RA is the one reported in 2017 by Nuzhdin and co-workers, that used Ag/γ−Al_2_O_3_ catalyst for one-pot reductive amination of aliphatic and aromatic aldehydes with nitroarenes under continuous-flow conditions [86]. This catalytic system was similar to the above reported Au/Al_2_O_3_ catalyst [82,83]. The catalyst used to fill the cartridge of the flow reactor was prepared by wet impregnation of γ-Al_2_O_3_ with silver nitrate, followed by calcination in air at 500 °C for 3 h. The as-prepared catalyst was formed by Ag NPs with a mean size of 5 nm dispersed onto the alumina matrix. However, before use, the catalyst was treated with H_2_ (3 MPa) at 110 °C for 1 h to be further reduced. Its catalytic activity was evaluated in the DRA under continuous flow conditions of nitroarenes and various aliphatic and aromatic aldehydes at 100 °C in toluene under H_2_ (3 MPa) to get fourteen *N*-substituted anilines containing alkyl, OH, OCH_3_, and even Cl, Br and C=C groups in good to excellent yields (Scheme 34). The accumulation of carbonaceous deposits on the catalyst was the main cause of catalyst deactivation. However, the activity of the spent catalyst could be restored completely through an oxidative treatment in air. The regeneration was carried out only once, but the morphology of the regenerated catalyst remained unchanged and no Ag NPs agglomeration occurred.

### 2.8. Other Metals (Group 11): Copper

**Scheme 35:**
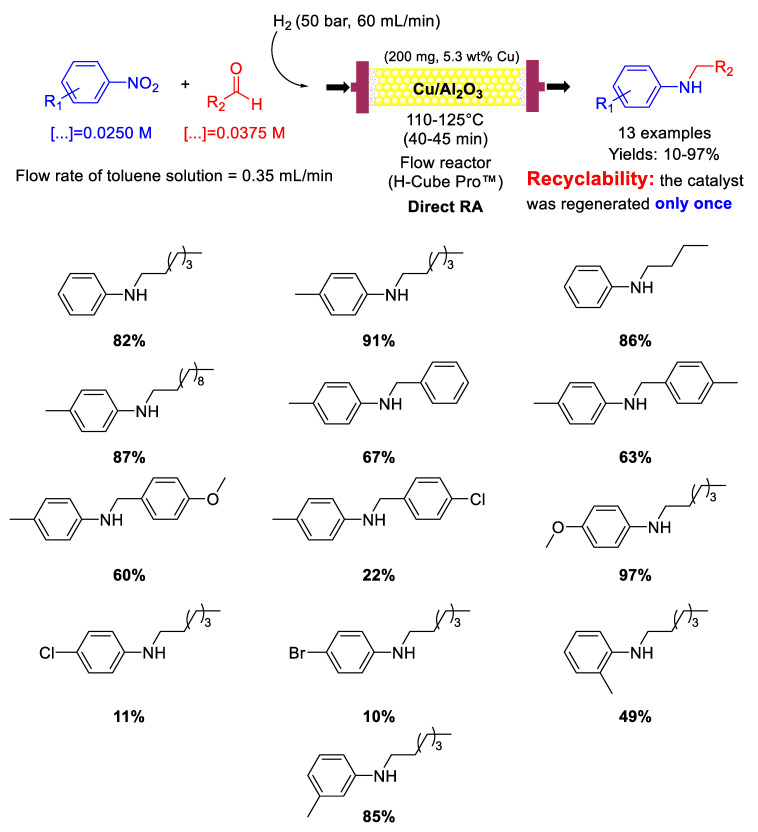
DRA catalyzed by Cu/γ-Al_2_O_3_ under flow conditions.

Following their research studies on DRA promoted by metal catalysts under continuous flow conditions, in 2017, Nuzhdin et al. prepared CuO/γ-Al_2_O_3_ pre-catalyst for the chemoselective hydrogenation of nitroarenes and one-pot reductive amination of aliphatic and aromatic aldehydes with different nitroarenes [87]. Before reaction the catalyst was reduced at 120 °C under 50 bar H_2_ for 60 min to get Cu/γ-Al_2_O_3,_ the real catalytically active species. The catalytic experiments were carried out in a continuous flow reactor, by using molecular hydrogen as reducing agent with a 1.5 molar excess of aldehyde with respect to nitroarene (because part of the aldehyde was reduced into alcohol) (Scheme 35). Indeed, methanol as the solvent was avoided, since in CH_3_OH the hydrogenation of the aldehyde was too fast, thus decreasing the yield into the desired secondary amine. Toluene resulted the best solvent. In the DRA of *n-*heptanal, introduction of electron-withdrawing substituents in *para*-position of the nitrobenzene ring decreased the reaction yields, while electron-donating substituents increased the final yields (opposite to what observed by Kalbasi and co-workers in their Pd system [61]). The observed effect of substituents suggested that the rate determining step of the overall reaction was the imine formation, because the electron properties of the substituents affected the nucleophilic features of the intermediate primary anilines. Various secondary amines were obtained in 10–97% yields (Scheme 35), and a comparison between Cu/γ-Al_2_O_3_ and the analogous Au/γ-Al_2_O_3_ [82,83] activities showed that the mass of the secondary amine (g) produced during 1 h per mass of metal (g), in Cu system was only 3.3–4.1 times lower than in Au one. Formation of carbonaceous deposits on the catalyst surface led to a slight deactivation of Cu/γ-Al_2_O_3,_ which was regenerated once by oxidative treatment in air at 330 °C for 2 h. The influence of different supports, such as γ-Al_2_O_3_, SiO_2_, and TiO_2_–SiO_2_ on the activity of Cu catalyst in DRA of *n-*heptanal with *p*-nitrotoluene under continuous flow conditions was deeply studied in a follow-up article [88], and it was demonstrated that Cu/γ-Al_2_O_3,_ was the most active catalyst among those considered. In the same article the authors proposed a heterolytic cleavage of dihydrogen in the catalytic cycles of the hydrogenation steps.

### 2.9. Transition Metals of the First Row of Group VIII: Iron

**Scheme 36:**
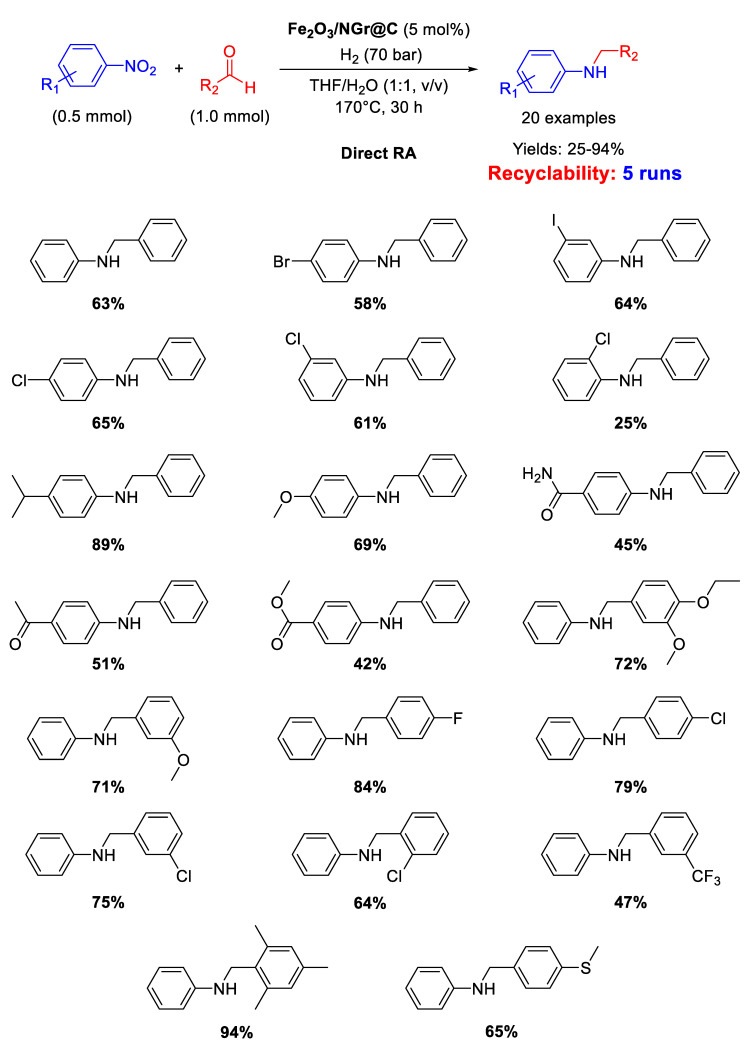
DRA catalyzed by Fe_2_O_3_/NG@C.

In recent years, Beller’s group has made many efforts in developing earth abundant metal based catalytic systems for DRA reaction. In 2014 they reported on the use of an iron oxide catalyst (Fe_2_O_3_/NG@C ) [77] for one-pot direct reductive amination of various benzaldehydes with different nitroarenes, using THF/H_2_O as the solvent [89] in quite harsh reaction conditions: T = 170 °C, 70 bar of dihydrogen, reaction time = 30 h (Scheme 36). The catalyst was obtained by reaction of Fe(OAc)_2_ and 1,10-phenanthroline, followed by addition of Vulcan XC72R as carbon support. The resulting suspension containing iron coated carbon powder was dried and pyrolyzed at 800 °C for 2 h under argon to get the final catalyst, namely Fe_2_O_3_/NG@C, which resulted core-shell structured with a core of Fe_2_O_3_ nanoparticles with size ranging from 2 to 100 nm (with some agglomeration) incorporated in a layer of N-doped graphene containing both pyridinic and pyrrolic nitrogen species. Doping a catalyst with nitrogen is a strategy largely used by Beller’s group and then followed also by other scientists, aiming at affecting the electronic features of the metals and at stabilizing the formed metal-based nanoparticles. Fe_2_O_3_/NG@C was recovered at the end of reaction and re-used for five cycles with a slight loss of activity.

**Scheme 37:**
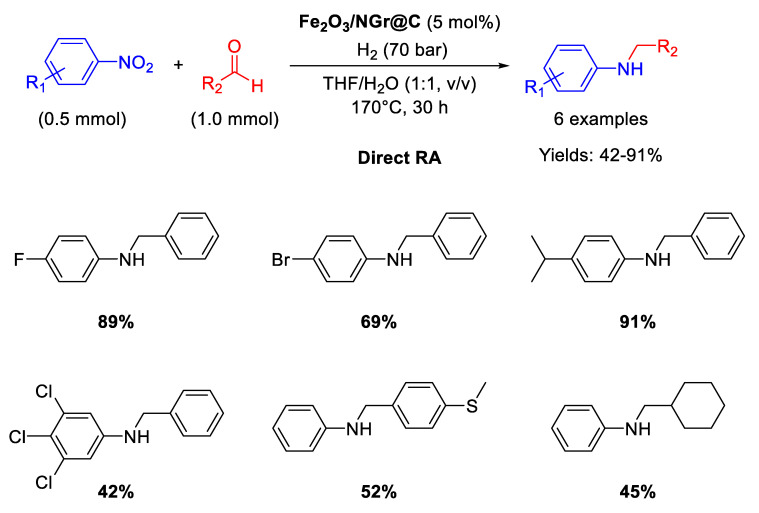
DRA catalyzed by Fe_2_O_3_/NGr@C.

In a follow-up article [90], Beller detailed the above reported protocol for both the synthesis of Fe_2_O_3_/NG@C and catalytic tests, isolating for all RA trials the products by flash chromatography (Scheme 37), obtaining for some examples yields slightly different from the ones reported in Scheme 36 for the same substrates.

### 2.10. Transition Metals of the First Row of Group VIII: Cobalt

**Scheme 38:**
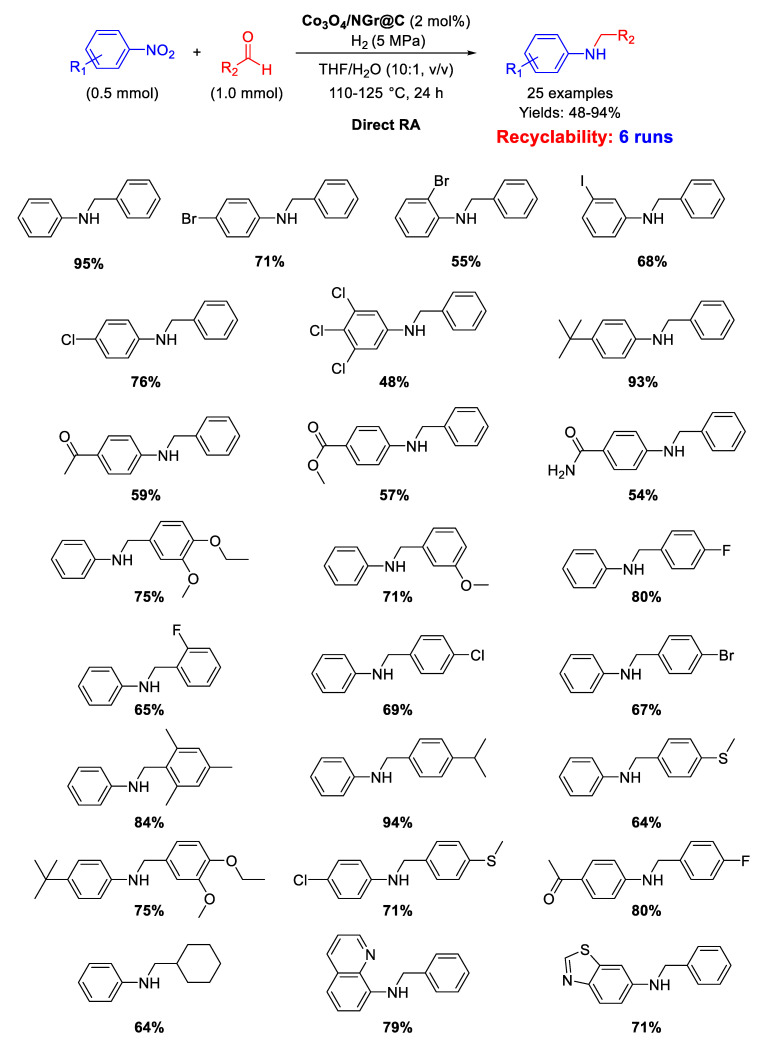
DRA catalyzed by Co_3_O_4_/NGr@C.

In 2013 Beller’s group synthesized the cobalt-based catalyst Co_3_O_4_/NGr@C (analogous to Fe_2_O_3_/NGr@C) by loading Vulcan XC72R carbon powder with a cobalt (II)-phenanthroline-chelate complex, and then pyrolyzing the obtained material at 800 °C for 2 h under Ar [76]. Co_3_O_4_/NGr@C was a core-shell nanocomposite constituted by a core of Co NPs and a shell of Co_3_O_4_ NPs with size in the range of 2–20 nm, forming agglomerates sizing 20–200 nm, coated in turn by a N-doped graphene layer. The so obtained heterogeneous catalyst was employed for DRA in THF/H_2_O under quite severe conditions: 110–125 °C, 50 bar of H_2_, reaction time = 24 h (Scheme 38) [91]. However, the designed catalytic system had the great advantage to tolerate the presence of other reducible groups, such as C=O, or halogen substituents (resisting to dehydrohalogenation) in the starting nitroarene ring or/and in the used benzaldehyde. This valuable peculiarity is a characteristic common to all Co-based catalytic systems for DRA proposed by Beller in subsequent works. Recyclability tests demonstrated that on passing from the first to the second run with the same catalyst, the yield dropped from 95% to 70% ca., then remaining constant at around 60% until the sixth cycle.

**Scheme 39:**
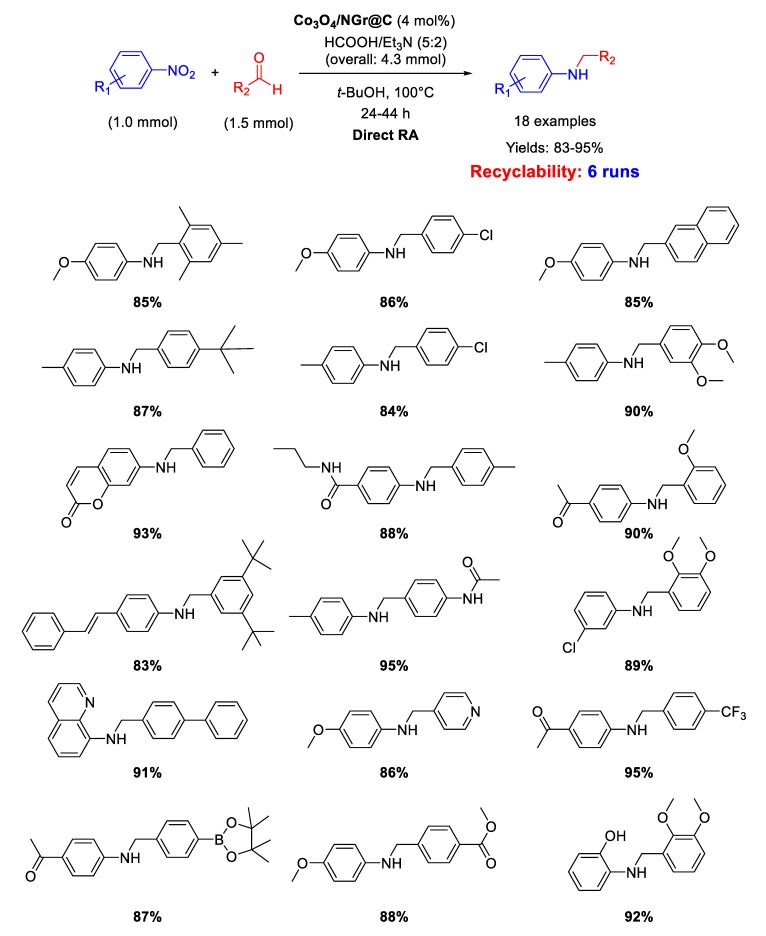
DRA catalyzed by Co_3_O_4_/NGr@C with HCOOH/Et_3_N.

In 2018 Jagadeesh and co-workers employed the same Co_3_O_4_/NGr@C material as catalyst for DRA, using in this case the mixture formic acid/Et_3_N (in 5/2 molar ratio), instead of H_2_, as the hydrogen donor (Scheme 39) [92]. The good yields obtained in the catalytic tests remained mainly constant also performing gram-scale reactions. The catalyst could be recycled up to five times demonstrating stability and reusability. This catalytic system was highly chemoselective, tolerating the presence of many reducible groups.

**Scheme 40:**
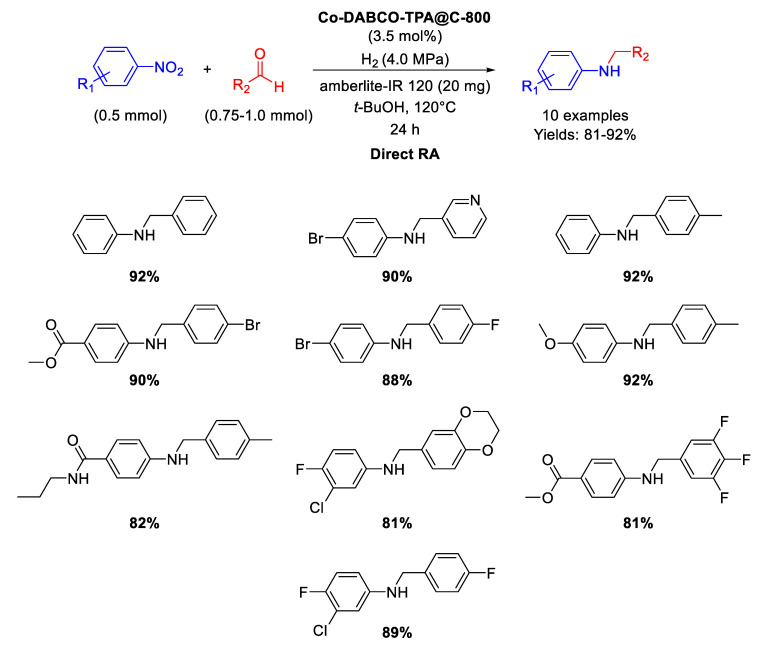
DRA catalyzed by Co-DABCO-TPA@C-800.

In 2017 Beller and co-workers synthesized MOF-derived cobalt nanoparticles catalytically active in direct RA reactions [93]. The catalyst was obtained by preparing a Co-diamine-dicarboxylic acid MOF precursor (namely Co-DABCO-TPA MOF, DABCO = 1,4-diazabicyclo [2.2.2]octane and TPA = terephthalic acid), which was in turn pyrolyzed on Vulcan XC72R carbon at 800 °C for 2 h under Ar, to get the final active nanomaterial (Co-DABCO-TPA@C-800), constituted by Co nanoparticles with diameters ranging from <5 to 30 nm, encapsulated in N-doped graphitic shells, supporting also single Co atoms. As in the previously reported work [91], some core-shell particles with cobalt oxide Co_3_O_4_ shells at metallic Co cores were also present. The new nanomaterial was tested as catalyst in the synthesis of a wide range of amines in good to excellent yields (Scheme 40) through DRA starting from different aldehydes with nitro compounds under 40 bar of molecular hydrogen at 120 °C in *tert*-BuOH in the presence of amberlite IR-120 for 24 h. The recyclability of the catalyst was tested in the synthesis of amphetamine, i.e. in the reaction between acetophenone and ammonia: the catalyst resulted recyclable for at least six runs without loss of activity and selectivity.

**Scheme 41:**
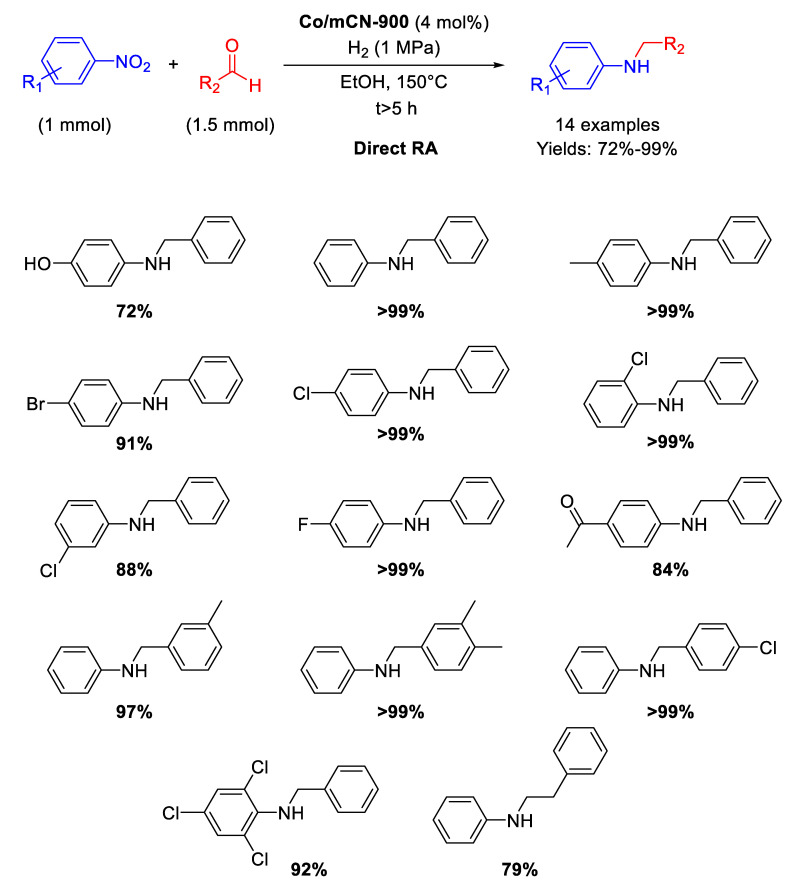
DRA catalyzed by Co/mCN-900.

Following the above mentioned strategy of using N-doped carbon support for synthetizing Co-based catalysts, in 2017 Cui and co-workers have described the preparation of a new nanomaterial (Co/mCN-900) by pyrolysis under N_2_ at 900 °C for 1 h of a mixture of melamine, polyacrylonitrile, and Co(NO_3_)_2_⋅6H_2_O [94]. In this way, a fluffy mesoporous carbon structure with high surface area and high nitrogen content was obtained. This N-doped carbon material supported dispersed accessible Co nanoparticle active sites (average size of 6 nm) and resulted an efficient catalyst in the N-alkylation of different nitrobenzenes with substituted benzaldehydes, allowing the synthesis of a wide range of aromatic amines with good yields under 1 MPa of H_2_, at 150 °C in ethanol (Scheme 41). The recyclability test was performed for the hydrogenation of nitrobenzene only (first step of the RDA) for six runs with the same catalyst. The catalytic system tolerated the presence of other reducible groups.

**Scheme 42:**
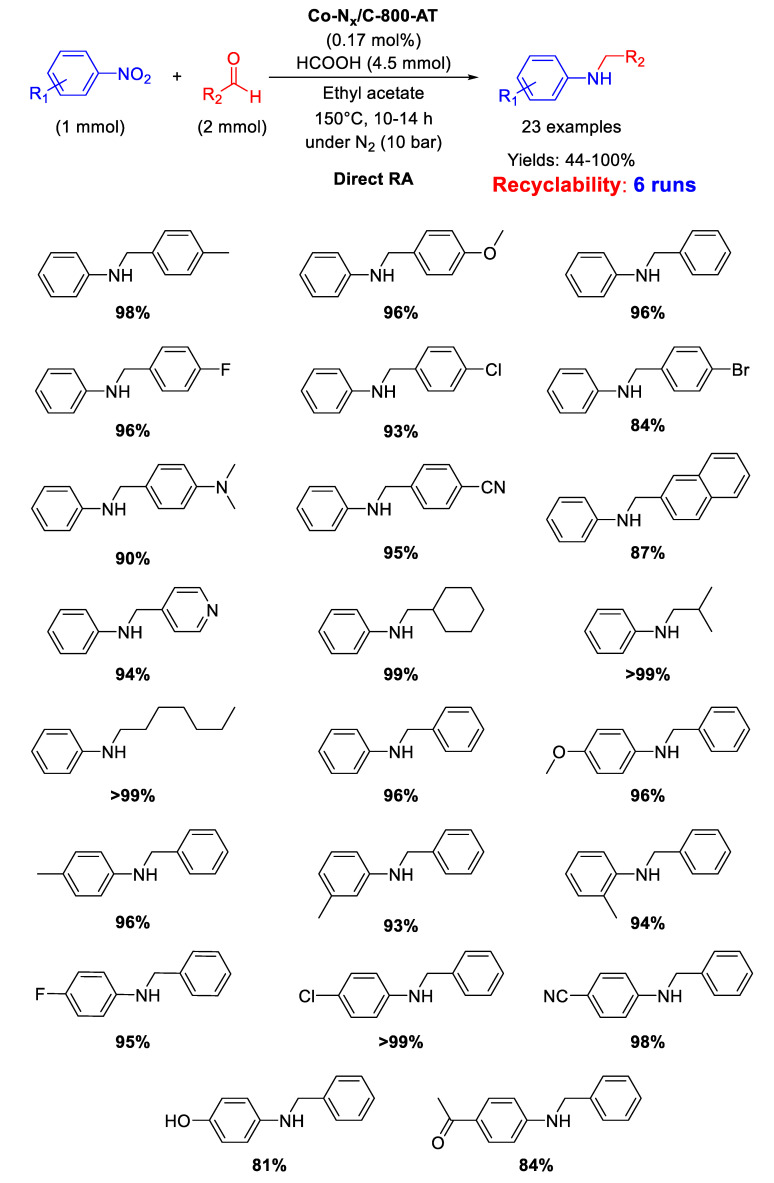
DRA catalyzed by Co-Nx/C-800-AT.

In 2017, Zhang and co-worker investigated the catalytic activity in DRA of a cobalt-based material, namely Co-Nx/C-800-AT [95], generated from the pyrolysis of cobalt phthalocyanine/silica composite at 800 °C under N_2_ and subsequent etching by HF to remove Co nanoparticles and silica template, affording a mesoporous polymer with mean pore size of 3.6 nm, containing Co-N_x_ catalytically active sites [96]. This strategy of synthesis, differently from the above reported ones, aims to remove all metal (or eventually metal oxide) nanoparticles, leaving a carbon-based support retaining just Co-N_x_ fragments, considered responsible for catalytic activity. As shown in Scheme 42, DRA was carried out at 150 °C in ethyl acetate using formic acid as hydrogen donor and a combination of different aldehydes and nitro compounds was well tolerated under reaction conditions. Moreover, the catalyst was active and stable during six runs without leaching of Co from the system. The catalytic system was highly chemoselective tolerating the presence of C=O, CN, and halogen atoms in the nitroarene ring.

**Scheme 43:**
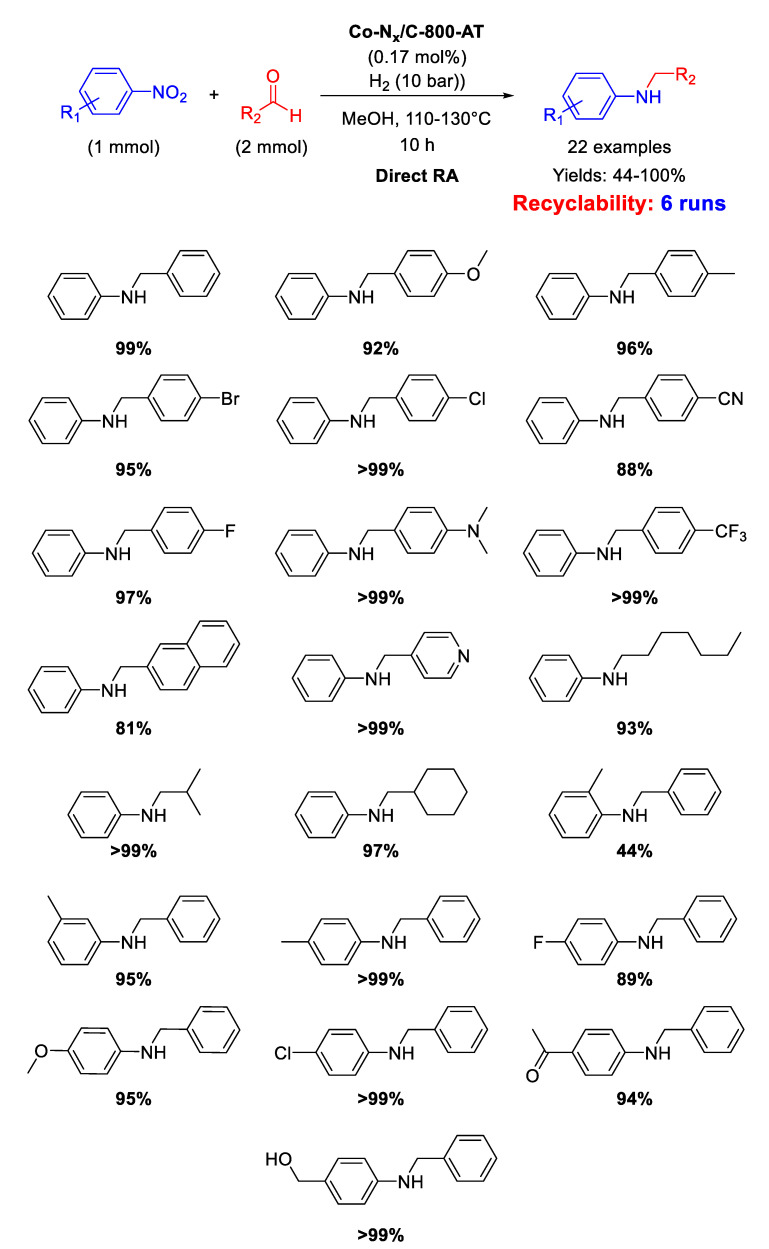
DRA catalyzed by Co-N_x_/C-800-AT under 10 bar H_2_.

In addition, the catalyst Co-N_x_/C-800-AT has been tested in the RDA reaction using H_2_ (10 bar), as the reductant, in methanol at 110 °C for 10 h [97]. Kinetic studies pointed out that, for the Co-N_x_/C-800-AT catalytic system, the rate-determining step of the overall reaction was the hydrogenation of the imine intermediate. For this reason, the steric hindrance of the substituents located in *ortho-*position in nitroarene and benzaldehyde substrates dramatically decreased the final yields towards the target molecule. Scheme 43 summarizes the wide scope of substrates explored. The catalyst was successfully recycled for six runs

**Scheme 44:**
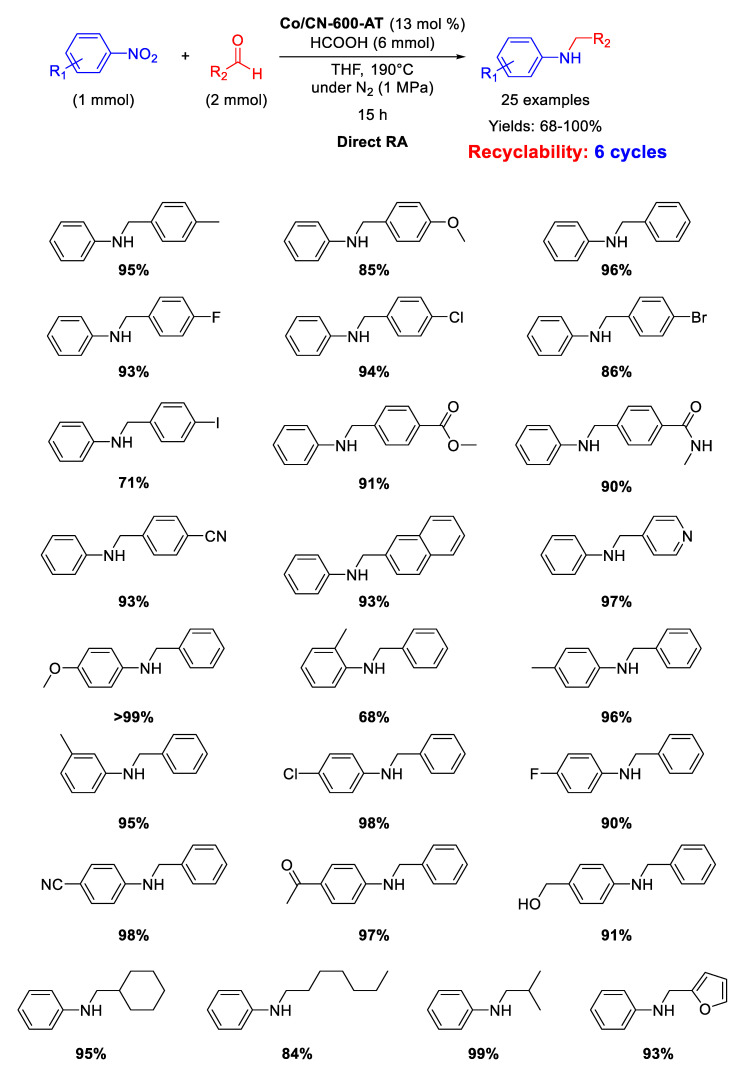
DRA catalyzed by Co@CN-600-AT.

On the basis of the above reported results, in the second part of 2017, Zhang’s group synthesized another catalyst by pyrolysis at 600 °C for 3 h under N_2_ of cobalt supporting zeolitic imidazolate-frameworks (ZIF-67)/graphene oxide (GO) composite (ZIF-67/GO), followed by acid-etching with H_2_SO_4_, aiming to remove Co NPs formed under calcination [98]. Differently from the Co-N_x_/C-800-AT material, in this case the acid washing was able to remove just the surficial metal nanoparticles of the new material and the resulting catalyst (Co@CN-600-AT) still contained Co NPs with an average diameter of 8.2 nm, embedded into the reduced graphene oxide phase (graphene oxide became reduced graphene oxide during calcination). Co@CN-600-AT was active, stable, and selective in the direct RA using formic acid as hydrogen source, in THF at 190 °C for 15 h (Scheme 44). The catalyst could be recycled for six times without losing its activity.

Following their studies on the synthesis of N-doped cobalt-based material, in 2017 Zhang’s group prepared another cobalt containing precursor, similar to the above reported ZiF-67/GO, but with silica in place of graphene oxide (ZIF-67/SiO_2_ composite). This cobalt containing material was pyrolyzed at 800 °C for 8 h under N_2_, and subsequently treated with hydrofluoric acidto remove surficial Co nanoparticles and silica template, yielding nitrogen-doped carbon-embedded Co nanoparticles with an average size of 6.3 nm (Co@CN-800) [99].

**Scheme 45:**
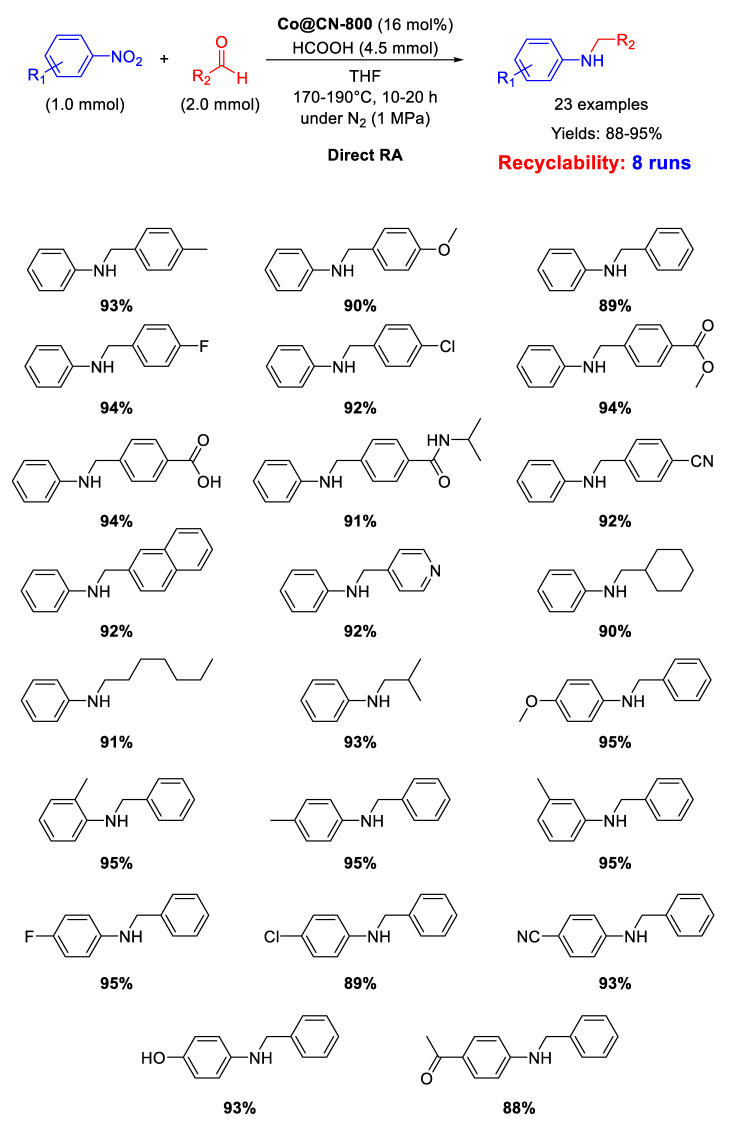
DRA catalyzed by Co@CN-800.

As shown in Scheme 45, Co@CN-800 catalyst was active and recyclable (for at least eight runs) in the direct reductive amination of various substrates using formic acid, as hydrogen donor, at 170–190 °C in THF.

**Scheme 46:**
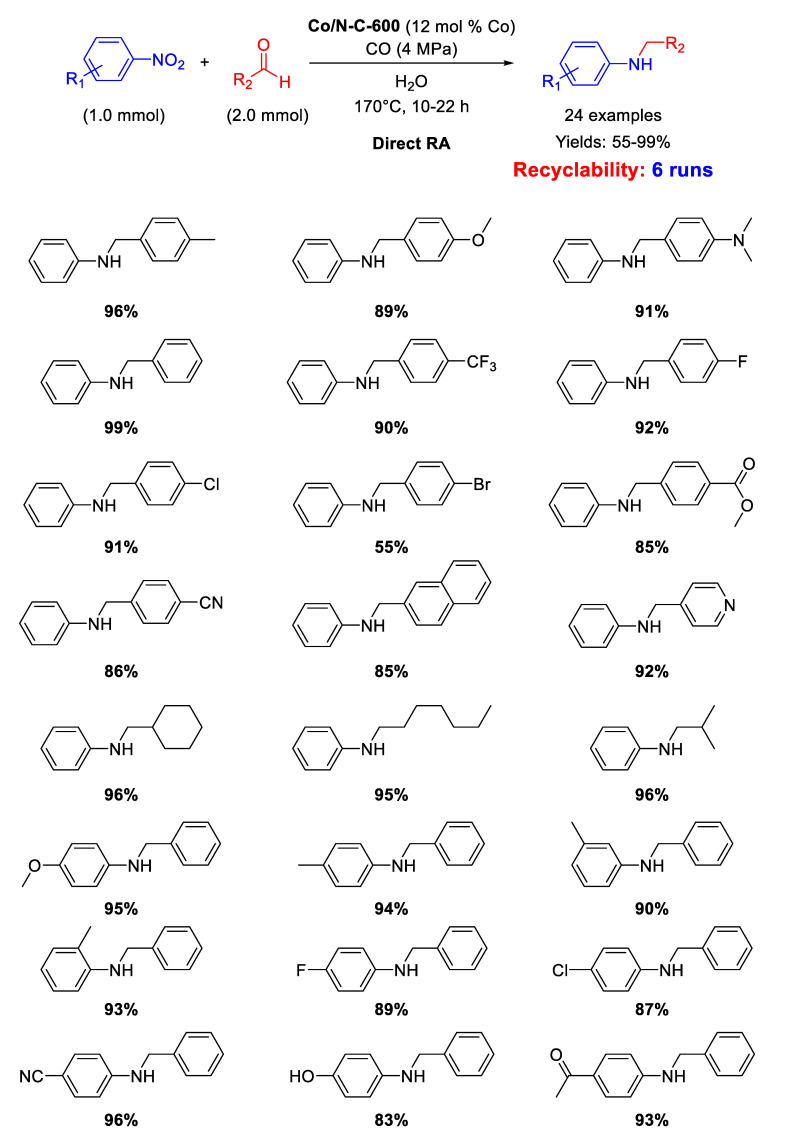
DRA catalyzed by Co/N-C-600 with CO/H_2_O.

Pyrolysis of ZIF-67 under N_2_ at 600 °C, followed by HF etching to remove Co NPs from the surface gave a new material (called Co/N-C-600) that was employed as catalyst for DRA. ZIF-67, as previously reported, consists of cobalt supported zeolitic imidazolate-frameworks, made up of cobalt cation and 2-methylimidazole ligand, obtained by simply mixing Co(NO_3_)_2_·6H_2_O with 2-methylimidazole in water. Co/N-C-600 contained Co-nanoparticles (with average diameter of 14 nm) embedded in N-doped carbon support and was active in the direct RA using, for the first time in this reaction, CO/H_2_O as hydrogen source [100]. The proposed catalytic cycle envisaged the formation of formate ion and the resulting transfer hydrogenation mechanism resembled the pathway observed with the use of acid formic as hydrogen donor. The presence of nitrogen in the N-doped material was of crucial importance because doping N served as base site to capture the H^+^ from water during the activation of CO/H_2_O, to generate the active species (a proton and a metal hydride). Several aldehydes were tested in the reaction with nitro compounds under the conditions experienced without loss of activity. Noteworthy, the strong interaction between cobalt nanoparticles and nitrogen atoms warranted a high stability of the catalyst for 6 recycling runs (Scheme 46).

**Scheme 47:**
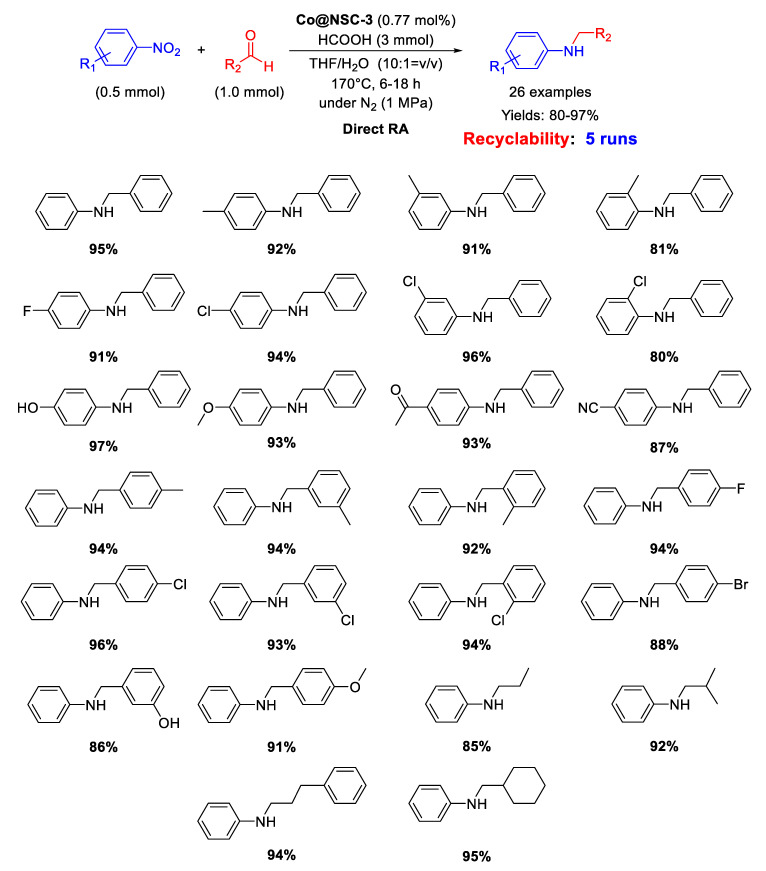
DRA catalyzed by Co@NSC-3.

In 2019, Guo’s group developed a N,S-dual-doped carbon material embedding Co nanoparticles with an average size of 2.01 nm, useful for catalyzing DRA with formic acid [101]. For the synthesis of the new catalyst, first the microwave assisted condensation of melamine and terephthalaldehyde was carried out in DMSO to give a Shiff base network (SNW-1, a porous organic polymer) incorporating sulfur impurities (due to the solvent used). Then, SNW-1 was impregnated with a cobalt salt and coated with glucose, and then pyrolyzed under inert atmosphere at 850 °C for 2 h. The resulting nanomaterial, Co@NSC-3 had a core-shell structure with cores of well-distributed cobalt NPs and N,S-doped carbon shells. Co@NSC-3 catalyzed the direct RA with formic acid as a hydrogen donor at 170 °C in THF/H_2_O. The system worked without a base and showed interesting activity, selectivity, and stability for the synthesis of several kinds of secondary amines (Scheme 47). Recyclability tests demonstrated that the catalytic system could be re-used for at least five runs without loss of activity and selectivity.

### 2.11. Transition Metals of the First Row of Group VIII: Nickel

**Scheme 48:**
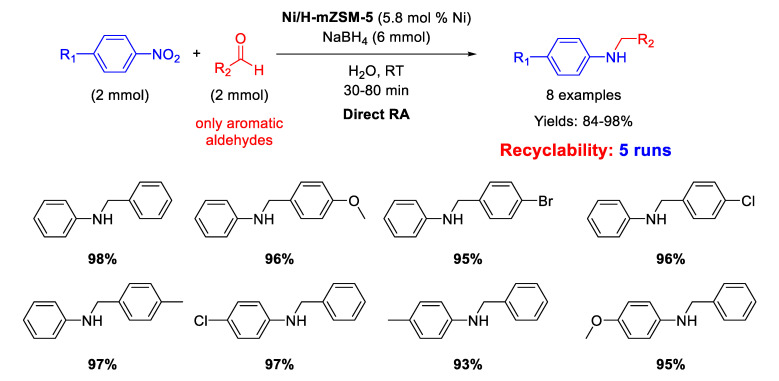
DRA catalyzed by Ni/H-mZSM-5.

In 2015 Kalbasi and Mazaheri [102] reported the synthesis of nickel nanoparticles (average size of 5 nm), supported on acid hierarchical zeolite (Ni/H-mZSM-5) with a microporous/mesoporous structure, as an acid-metal bifunctional catalyst. The catalytic activity of Ni/H-mZSM-5 was tested in one-pot RA of aromatic aldehydes with nitroarenes at room temperature, by using sodium borohydride as reducing agent and water as the solvent (Scheme 48). The simultaneous presence of acid and metal in the catalytic structure promoted a high activity and reusability of the heterogeneous catalyst in water under mild conditions for at least five runs, with a slight reduction of activity (the yield dropped from 98% of the first run down to 73% of the fifth cycle). The efficiency of the Ni catalytic system prompted the same authors to replace Ni for Pd, preparing the analogous Pd/H-hierarcal ZSM-5 already discussed in [61].

**Scheme 49:**
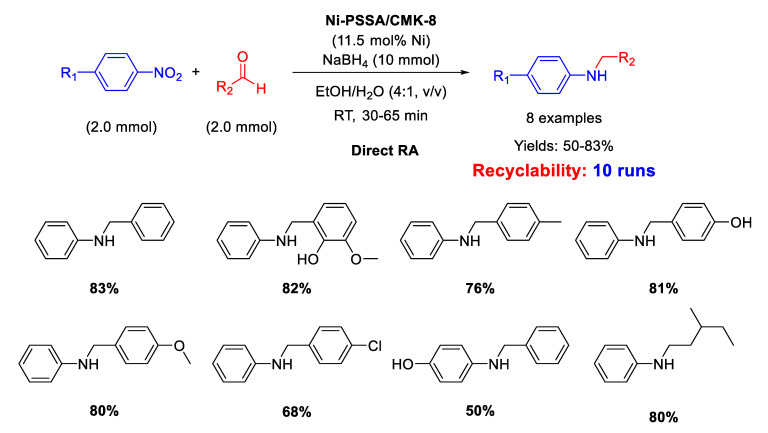
DRA catalyzed by Ni- PSSA/CMK-8.

On the basis of the above reported results, Kalbasi and coworker [103] developed a new heterogeneous catalyst constituted by ordered mesoporous carbon CMK-8 supporting both Ni nanoparticles (size ranging from 2 to 6 nm) and polystyrene sulfonic acid (PSSA) chains, thus having a dual (metal and acid) functionality. The new heterogeneous bi-functional catalyst, Ni-PSSA/CMK-8 was similar in the concept to the previously reported Ni/H-mZSM-5. The difference between the two catalysts was in the solid support: while hierarchical ZSM-5 is an inorganic solid acid with a relatively low surface area, PSSA/CMK-8 composite is an organic solid acid with a higher surface area, thus potentially more active. The new catalyst was tested in direct RA of aldehydes under mild reaction conditions and short reaction time in ethanol/water as solvent (Scheme 49). Recyclability tests demonstrated that the catalyst could be used for at least ten runs with slight metal leaching into solution and small decrease of yield (83% in the first run; 68% in the 10^th^ cycle). For DRA carried out with Ni-PSSA/CMK-8 the authors observed the same substituent electronic effects registered with Pd/H-hierarchical ZSM-5 catalytic system [61], that is: the reaction rate was improved by the presence of electron withdrawing and electron donating substituents in nitrobenzene and benzaldehyde rings, respectively. In addition, to get insights into the mechanism pathway, Kalbasi and coworker mixed aniline, benzaldehyde, and Ni-PSSA/CMK-8 in EtOH/H_2_O in the absence of the reductant and observed 40% yield in imine (no secondary amine formation), but when they added to the mixture the appropriate amount of NaBH_4_, they registered 83% yield in the target molecule (secondary amine), and not 40% as they thought to find. On the basis of this observation, the authors claimed for their catalytic system the presence of an additional mechanism for the imine formation, passing through the reaction between benzyl alcohol (eventually formed under reaction conditions, but not detected by the authors) and aniline, to give imine, subsequently reduced to secondary amine. However, this speculation is not convincing because the condensation between benzaldehyde and aniline is an equilibrium reaction [36], whose balance is shifted towards the imine product once the latter is subtracted from the system, due to its hydrogenation. Therefore, it was not surprising that the yield into secondary amine increased when the imine was removed from the equilibrium.

**Scheme 50:**
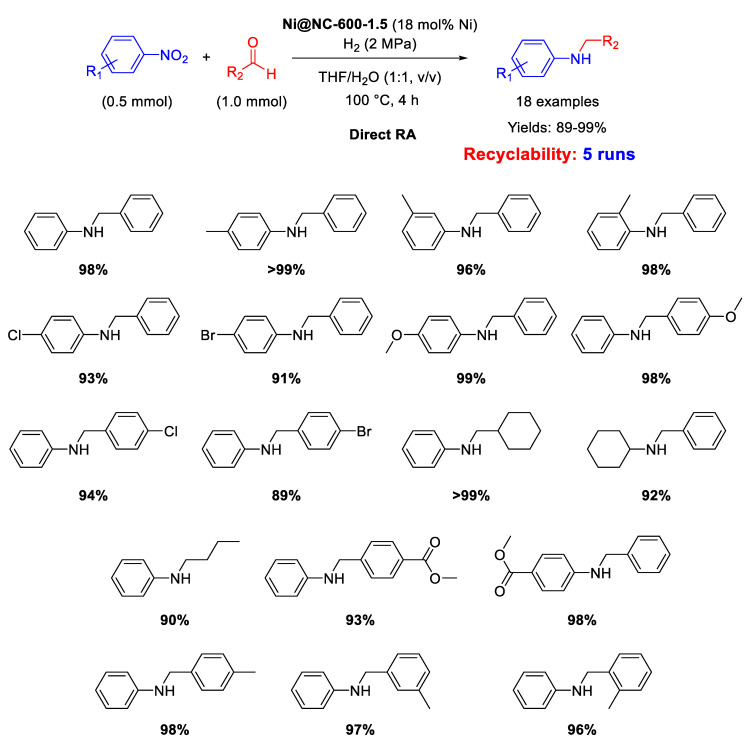
DRA catalyzed by Ni@NC-600-1.5.

In 2019 Wang and Chen prepared a MOF-derived catalyst with nickel nanoparticles (6–7 nm in size) uniformly embedded in nitrogen doped carbon shells [104], following roughly the strategy already introduced by Beller and discussed in the above reported paragraphs. In details, the new catalyst was obtained by mixing 2-aminoterephthalic acid and polyvinylpyrrolidone with a nickel(II) salt in DMF/water, thus obtaining a green solid precipitate (Ni-MOF-NH_2_), which was pyrolyzed at 600 °C for 1.5 h under Ar to get the final Ni@NC-600-1.5 catalyst. The new catalyst resulted active and selective for at least five runs in direct RA under H_2_ (2 MPa) at 100 °C in THF/H_2_O (Scheme 50). The catalyst possessed magnetic features which allowed its easy removal from the reaction mixture by an external magnet, and its subsequent recycling for five runs without loss of activity and recyclability. XRD analyses excluded the formation of NiO, while N-doping promoted the dispersion of Ni nanoparticles and enhanced their ability to activate H_2_ through heterolytic cleavage, further facilitated by the presence of pyridinic N found in the carbon shells.

**Scheme 51:**
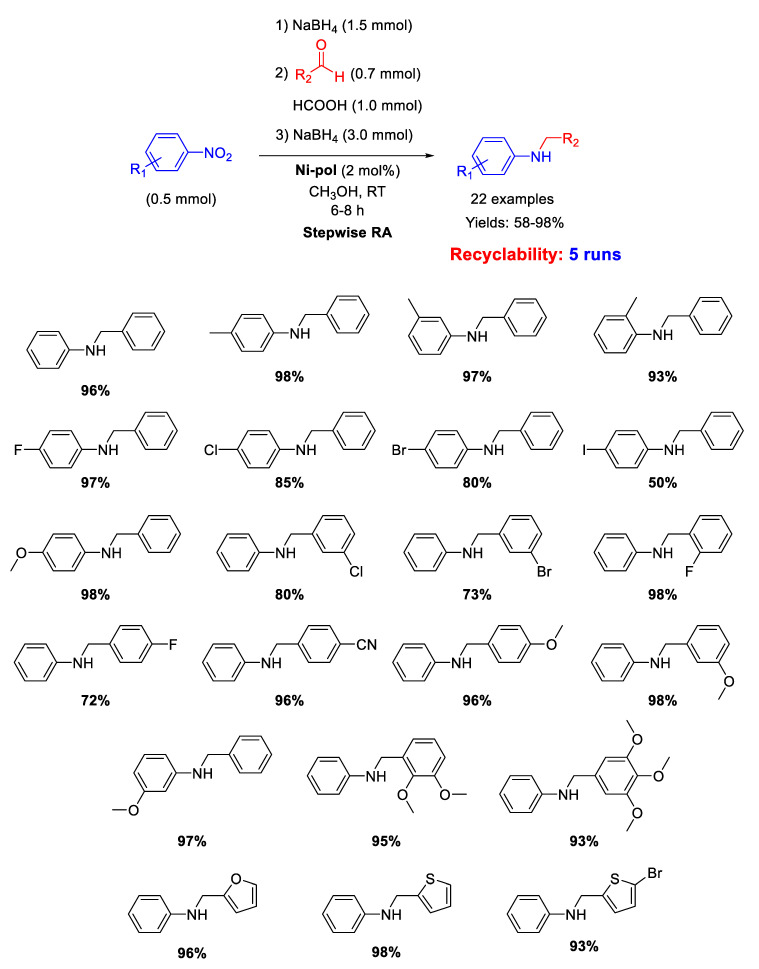
Stepwise RA catalyzed by Ni-pol.

Another example of N-doped organic matrix supporting Ni NPs as efficient catalyst for RA reaction has been reported in 2019 by Dell’Anna and co-workers [105]. Indeed, on the basis of the good results obtained with the already discussed Pd-pol [53], the strategy of synthesis followed for Pd-pol was transferred for the synthesis of Ni-pol, wherein the noble metal Pd was replaced by the earth abundant nickel. The polymer has been prepared by co-polymerizing the metal containing monomer [106] Ni(AAEMA)_2_ [AAEMA^−^ = deprotonated form of 2-(acetoacetoxy) ethyl methacrylate] with *N*,*N*-dimethylacrylamide and *N,N*’-methylenebisacrylamide [107] and submitting it to calcination at 300 °C under N_2_, obtaining a N-containing organic polymer (Ni-pol) with a homogeneous distribution of cubic nanocrystals of Ni of average cross section value of 35 nm [108]. Ni-pol was tested in the stepwise RA of various benzaldehydes with different nitroarenes in methanol at room temperature, using NaBH_4_ as the reductant (Scheme 51). The addition of few drops of formic acid was necessary in the condensation step to promote the formation of the imine intermediate. Twenty-two secondary amines have been synthetized with Ni-pol catalytic system, some of them containing halogen substituents (no hydro-dehalogenation occurred) or reducible group (-CN). Ni-pol was recovered at the end of reaction and used for five subsequent runs without loss of activity and selectivity.

**Scheme 52:**
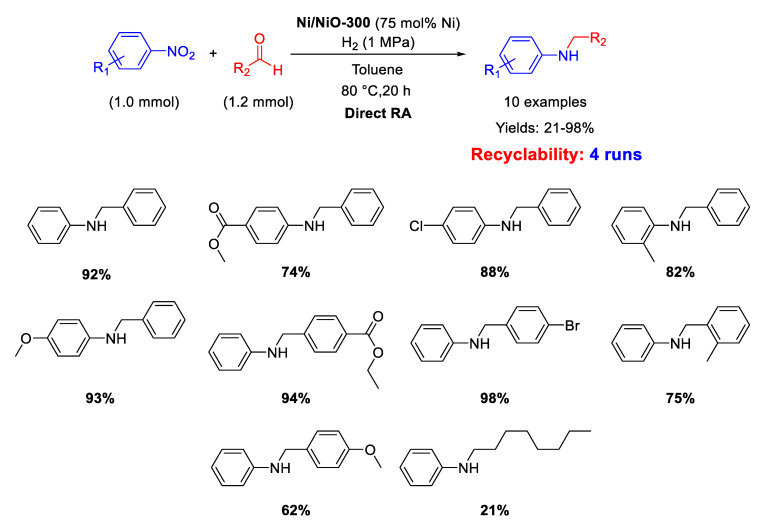
DRA catalyzed by Ni/NiO-300.

In 2020, Hara and co-workers prepared Ni/NiO-300 by partial reduction of NiO at 300 °C with H_2_ and employed it as heterogeneous catalyst for one-pot reductive amination of benzaldehydes with different nitroarenes under dihydrogen (1 MPa) in toluene for 20 h (Scheme 52) [109]. The catalyst could be reused for four cycles without catalytic losses. Scanning Electron Microscopy (SEM) and XPS analyses showed that Ni/NiO-300 was constituted by Ni particles with size of about 0.5–3 μm coated with a layer of NiO, which remained the same after catalytic cycles. The metal amount used is very high for a catalytic reaction.

### 2.12. MoS_2_ Catalyst

**Scheme 53:**
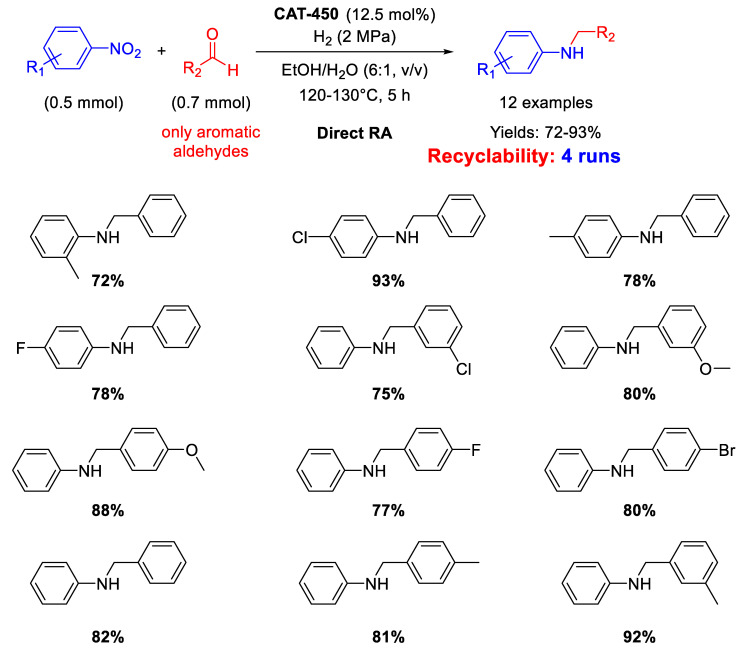
DRA catalyzed by CAT-450.

There is just one example reported in 2019 by Zhang et al. that used defective MoS_2_ with sulfur vacancies for catalysing RDA to prepare secondary amines in excellent yields. MoS_2_ was subjected to thermal treatment at 450 °C under nitrogen atmosphere (to get the so-called CAT-450) to improve its catalytic activity due to the sublimation of MoS_2_ and sulfur [110]. Since sublimation of sulfur is faster than sublimation of MoS_2_, large sulfur vacancies area on MoS_2_ surface formed in CAT-450. These vacancies could effectively adsorb and activate dihydrogen, nitro compounds, and imines, for efficiently catalysing DRA of various nitroarenes and benzaldehydes in EtOH/H_2_O, at 120–130 °C under 2 MPa H_2_ for 5 h (Scheme 53). CAT-450 could be recycled without decrease of its catalytic performance after the fourth run.

### 2.13. Heterobimetallic Catalysts

Recently, many efforts have been devoted to designing heterobimetallic catalytic systems active for RA, aiming at conjugating the properties of two different metals, hoping in a synergic action. In fact, using simultaneously two metals active in two different steps of the RA process could in principle improve the final yield towards the target product. In addition, a metal of a heterobimetallic catalyst could lower the extent of side reactions (such as reduction of aldehyde, C-N bond hydrogenolysis, etc.) deriving from the use of the other metal alone. Moreover, as for monometallic catalysts, the goodness of a heterobimetallic catalytic system could be enhanced by choosing a proper insoluble support with acidic, basic, or magnetic features.

#### 2.13.1. PdAg

**Scheme 54:**
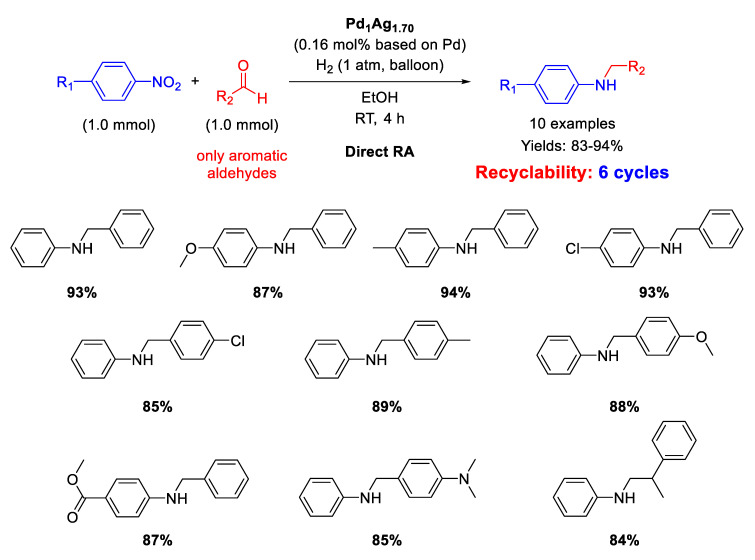
DRA catalyzed by Pd_1_Ag_1.7_.

The first application of heterobimetallic nanoparticles for DRA from nitroarenes and aldehydes was reported in 2013 by He, Li and co-workers [111]. By co-reduction of Pd(acac)_2_ and AgOOCCF_3_ using a solution of borane-tert-butylamine adduct in oleylamine, they were able to synthesize a series of Pd_1−x_Ag_x_ (x = 0–1) nanoparticles, among which Pd_1_Ag_3_ NPs, with mean particle size of ca. 3 nm was found as optimal catalyst for DRA in terms of activity and selectivity under mild conditions (1 atm H_2_ and RT). The exact Pd/Ag ratio was determined by inductively coupled plasma atomic emission spectroscopy (ICP-AES) analysis, and the catalyst was renamed as Pd_1_Ag_1.7_. Ten secondary amines were obtained in good to excellent yields (83–94%) with a good functional group compatibility (Scheme 54). The catalyst could be easily recovered by centrifugation and reused at least five times without loss of activity. Investigations by TEM revealed no observable structural change in the dispersion of the spent catalyst with respect to the fresh one.

**Scheme 55:**
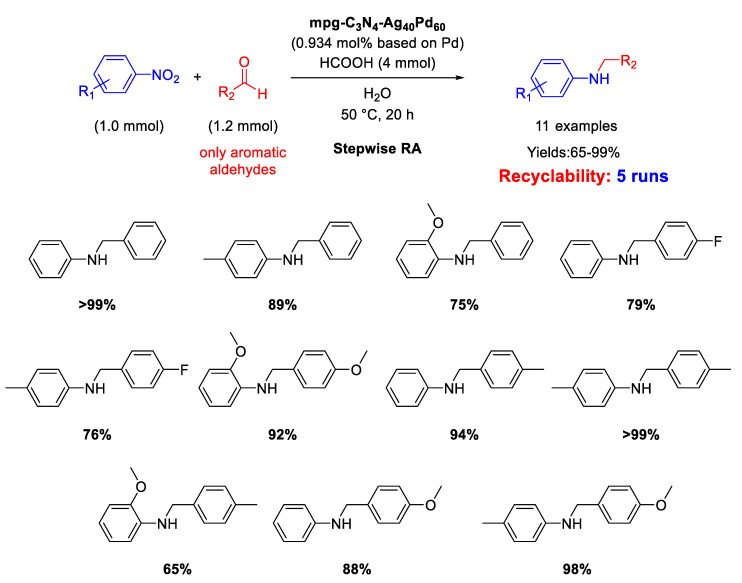
DRA catalyzed by mpg-C_3_N_4_/Ag_40_Pd_60_.

Another heterobimetallic catalyst based on silver and palladium for RA from nitroarenes and aldehydes was proposed in 2018 by Metin and co-workers [112]. Taking advantage of their previous experience in the synthesis of AgPd alloy NPs by one-pot co-reduction of silver(I) acetate and palladium(II) acetylacetonate in the presence of oleylamine and oleic acid as surfactants in hot 1-octadecene [113], the authors were able to support, via. a liquid phase self-assembly method, these NPs (2.5 ± 0.5 nm) on high-surface area and commercially available mesoporous graphitic carbon nitride (mpg-C_3_N_4_) obtaining the mpg-C_3_N_4_/Ag_40_Pd_60_ catalyst. A protocol in harmony with the green chemistry rules was developed with this catalyst. In fact, it promoted a stepwise RA of arylaldehydes with nitroaromatics utilizing formic acid as hydrogen source and water as solvent (Scheme 55) furnishing eleven *N*-benzylanilines in moderate to excellent yields (65–99%). The proposed catalytic system could be reused at least 4 times without loss of activity, and investigations by TEM and powder XRD on recycled catalyst revealed that there was almost no change with respect to the initial morphology and crystal structure of fresh mpg-C_3_N_4_/Ag_40_Pd_60_ catalyst.

#### 2.13.2. PdAu

**Scheme 56:**
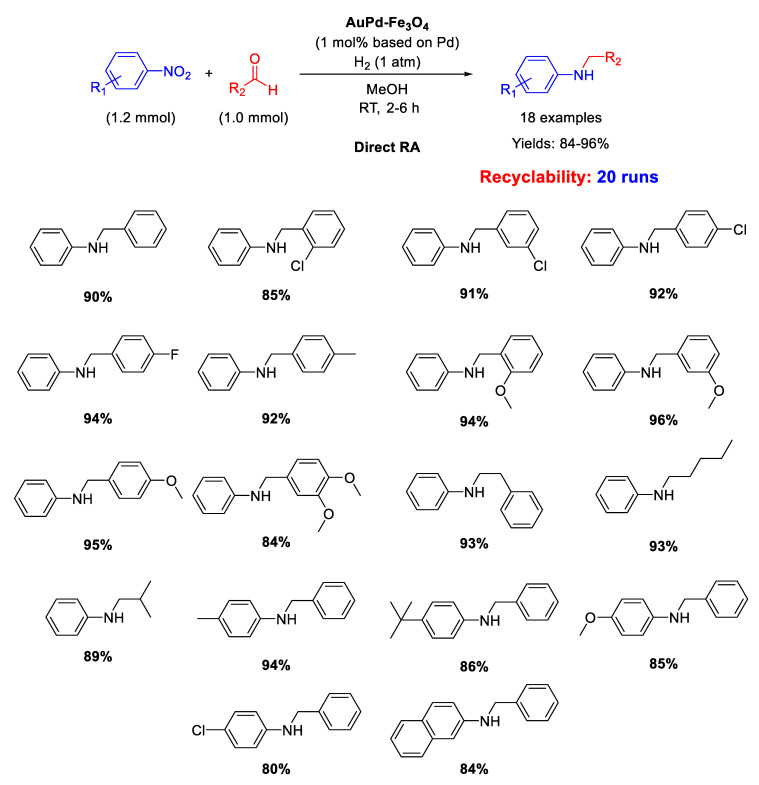
DRA catalyzed by AuPd–Fe_3_O_4_.

In 2018 Kim and co-workers prepared an AuPd alloy supported on magnetic Fe_3_O_4_ nanoparticles with mean particle size of ca. 5 nm (AuPd–Fe_3_O_4_) from PdCl_2_ and AuCl_3_·3H_2_O in presence of polyvinylpyrrolidone (PVP) and subsequent reduction with NaBH_4_ [114]. Investigations through X-ray photoelectron spectroscopy (XPS) of the oxidation states and electronic properties on AuPd–Fe_3_O_4_ surface confirmed the formation of an alloy with Au and Pd metals that remained unchanged in these properties after a catalytic cycle. AuPd–Fe_3_O_4_ nanocatalyst promoted selective DRA from a variety of nitroarenes and aryl or alkyl aldehydes under 1 atm of H_2_ at RT in methanol (Scheme 56). The selective syntheses of eighteen secondary amines in good to excellent yields were obtained and no *N*-debenzylation was remarkably observed in the case of reactions involving aryl aldehydes. Due to well-known magnetic property of the support, the separation of the AuPd–Fe_3_O_4_ catalyst could be rapidly realized by a small neodymium magnet and the catalyst could be recycled for further nineteen recycles without an appreciable loss of activity and significant metal leaching (only 7% loss of the initial content of gold and palladium). Under the optimized reaction conditions, the catalytic activity was maintained until the 13^th^ reaction cycle and a slight decrease of yield was observed from the 14^th^ to 16^th^ cycles. However, raising the reaction time from the 17^th^ to 20^th^ cycles the yields returned to the average values found in previous cycles. Thanks to the aid of accurate investigations by TEM and SEM, the authors were able to find that the modest metal leaching would not be the main reason for the reduced catalytic activity but that this was caused by a distinctive agglomeration of AuPd nanoalloy on Fe_3_O_4_ support after the 16^th^ and 20^th^ reaction cycle (see the Supporting Information of [114] for TEM and SEM images of the used catalyst).

Liang and co-workers reported a series of catalysts for DRA, based on Au–Pd alloys well dispersed within amine functionalized metal-organic framework (MOF, UiO-66-NH_2_) [115]. However, only Au–Pd alloy nanoparticles with low Pd content (Au-Pd_0.03_@UiO-66-NH_2_) exhibited high selectivity for DRA with benzaldehyde and nitrobenzene, and thus the sole synthesis of *N*-benzylaniline in 93% yield was obtained in cyclohexane as solvent at 50 °C under 1.0 MPa of H_2_.

#### 2.13.3. PdPt

**Scheme 57:**
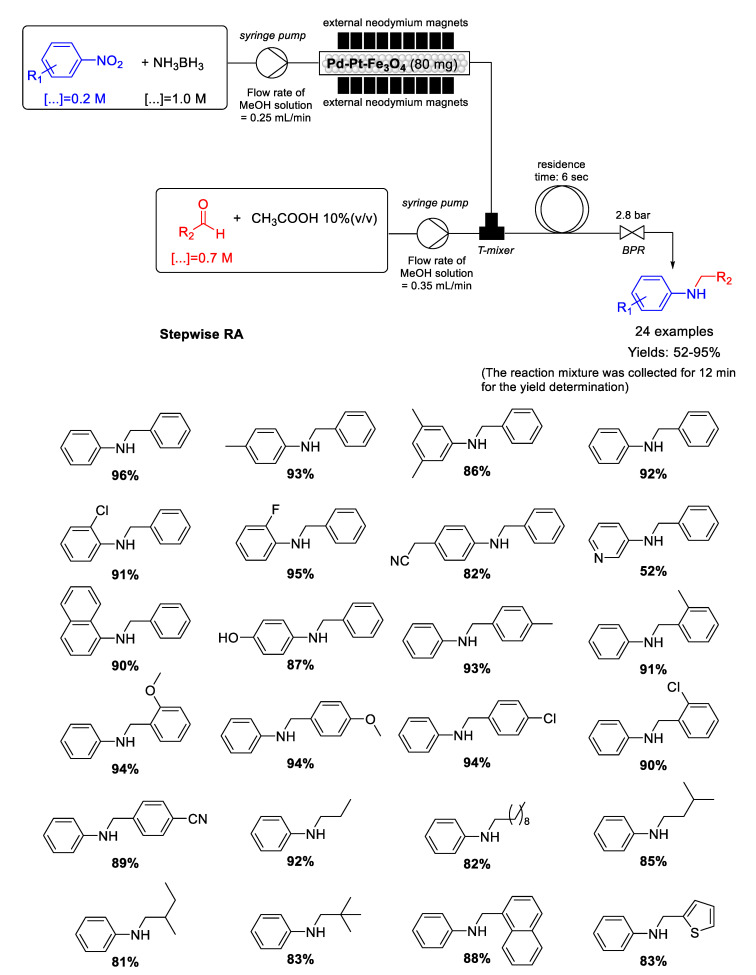
Stepwise RA catalyzed by Pd−Pt−Fe_3_O_4_ under continuous flow conditions.

In 2016 Kim group reported the synthesis of heterogenized bimetallic Pd−Pt−Fe_3_O_4_ nanoflakes via. the simultaneous reduction of PdCl_2_ and K_2_PtCl_4_ in presence of PVP as a dispersant and ethylene glycol acting as both solvent and reductant, followed by deposition of the two metal NPs on commercially available Fe_3_O_4_ nanocrystals in a simple one-pot solution phase hydrothermal process [116]. Fe_3_O_4_ support was decorated with well-dispersed Pd–Pt NPs having diameters ranging from 5 to 8 nm. Pd−Pt−Fe_3_O_4_ proved to be a very active and magnetically recyclable catalyst for chemoselective reduction of several nitroarenes with the use of ammonia borane (NH_3_BH_3_) as a hydrogen source. These characteristics prompted Kim’s and Park’s groups to explore the use of Pd−Pt−Fe_3_O_4_ as magnetically catalyst for developing a very fast stepwise RA from nitroarenes and aldehydes at room temperature using NH_3_BH_3_ as reducing agent in a continuous flow system (Scheme 57) [117]. The peculiarity of the flow system developed by these authors is that the catalyst was directly fixed in a simple perfluoroalkoxy alkanes (PFA) tubing with the aid of commercially available permanent neodymium magnets without the use of a special cartridge or column. Obviously, the fixation of magnetic nanoflakes of Pd-Pt-Fe_3_O_4_ in tubes obtained in this way resulted reversible by removing the neodymium magnets. The selective syntheses of twenty-four secondary amines in moderate to excellent yields were obtained and, moreover, a long-term (24 h) effectiveness experiment using nitrobenzene and benzaldehyde, showed an average yield of 93% (12.2 g) in *N*-benzylaniline without Pd and Pt leaching.

#### 2.13.4. CoRh

**Scheme 58:**
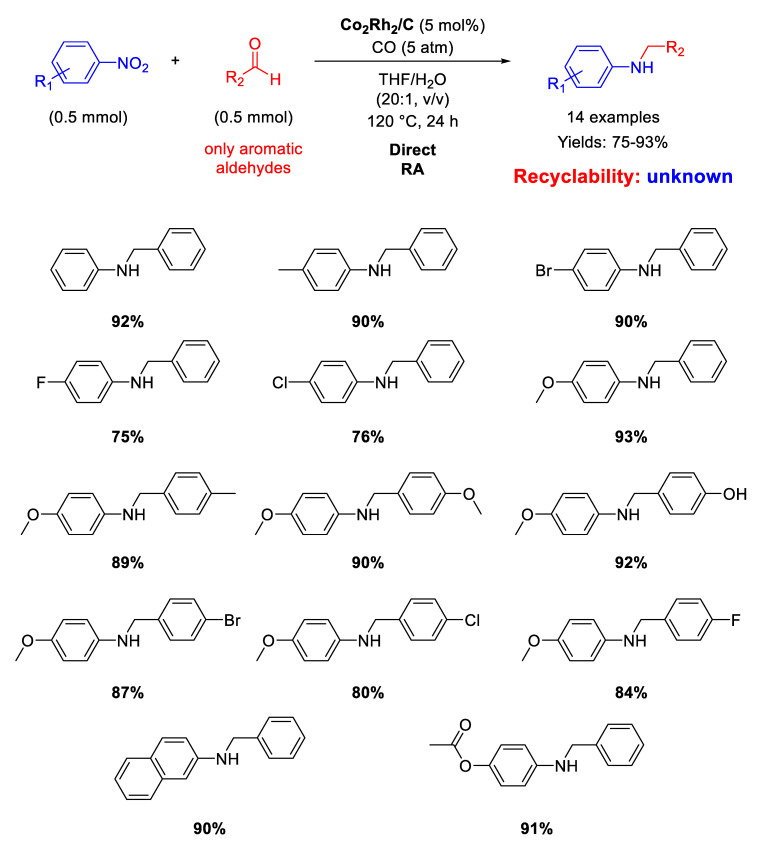
DRA catalyzed by Co_2_Rh_2_/C.

In 2015 the Chung group developed cobalt/rhodium heterobimetallic nanoparticles immobilized on charcoal Co_2_Rh_2_/C as catalyst for RA from aldehydes with nitroarenes under 5 atm pressure of carbon monoxide without an external hydrogen source, which is produced via a water gas-shift reaction thanks to water both added and generated in situ [118]. Co_2_Rh_2_ nanoparticles were obtained by reacting cobalt and rhodium carbonyl cluster Co_2_Rh_2_(CO)_12_ in presence of oleic acid and trioctylphosphane oxide in hot *ortho*-dichlorobenzene and were eventually supported on flame-dried charcoal. The so-obtained Co_2_Rh_2_/C catalyst promoted the synthesis of fourteen *N*-benzylanilines in good to excellent yields in THF/H_2_O mixture at high temperature (Scheme 58).

**Scheme 59:**
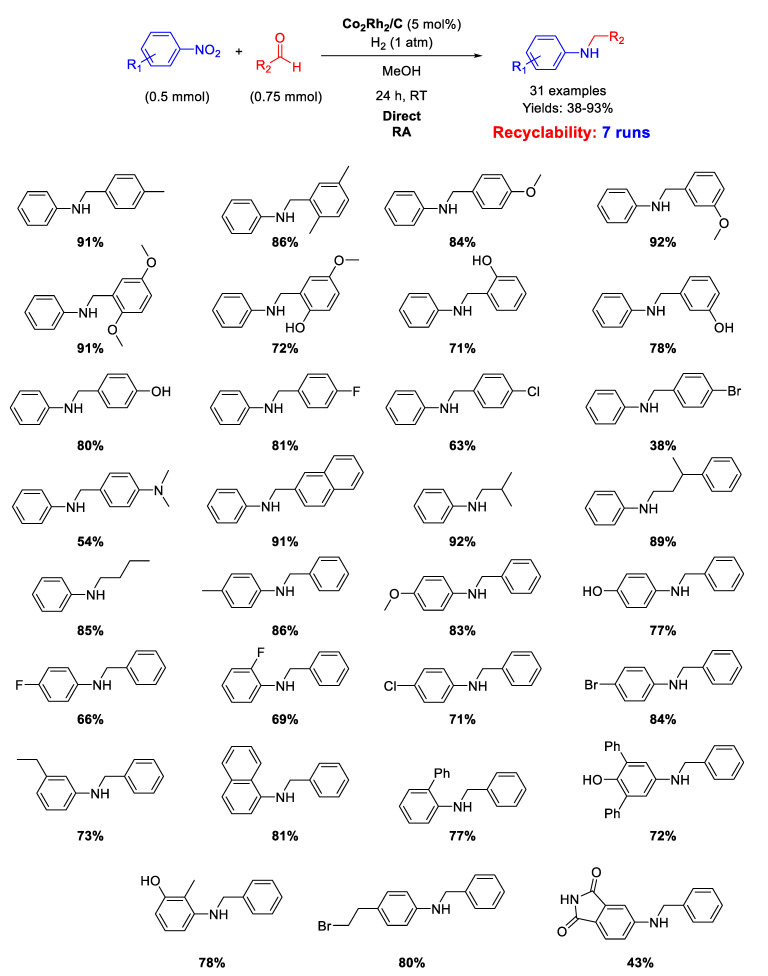
DRA catalyzed by Co_2_Rh_2_/C under H_2_.

Two years later [119], the same group reported that the heterogeneous Co_2_Rh_2_/C catalyst also showed excellent activity and good selectivity for one-pot DRA of aldehydes with nitroarenes in methanol under mild conditions (1 atm H_2_ and 25 °C) without any additives (Scheme 59). A very wide scope of reaction was demonstrated by reporting the synthesis in moderate to excellent yields of thirty-one secondary amines with very broad functional group compatibility. The proposed procedure was also scaled up to the gram scale, and the Co_2_Rh_2_/C catalyst could be easily recovered by centrifugation and recycled at least six times without loss of activity. No investigations on spent catalyst morphology were reported.

#### 2.13.5. FeNi

**Scheme 60:**
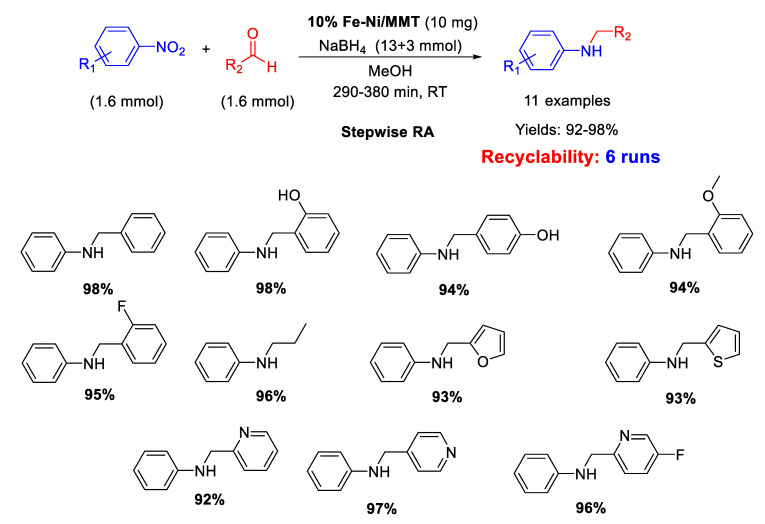
Stepwise RA catalyzed by 10% Fe–Ni/MNT.

In 2020 Chikate et al. immobilized non-noble Fe–Ni bimetallic nanoparticles (10%_w_) on montmorillonite (MMT) generating a bifunctional nanocomposite 10% Fe–Ni/MNT, which was tested as catalyst for a stepwise RA of aldehydes with nitroarenes using NaBH_4_ as hydrogen source (Scheme 60) [120]. Eleven secondary anilines were obtained in excellent yields and the catalytic system could be recycled at least five times without loss of activity. XPS studies carried out with fresh and recovered nanocomposite showed that surface properties of catalyst remained unaltered during reductive amination.

Finally, carbon-supported Fe–Ni alloy nanoparticles for DRA in a continuous fixed-bed reactor were already reported in 2015 by Esposito and co-workers. In this work the authors carried out DRA from nitrobenzene and benzaldehyde (3 equiv with respect to nitrobenzene) under very harsh conditions (125 °C and 10 bar of H_2_) achieving *N*-benzylaniline in 83% yield [121].

## 3. Conclusions

By reviewing results obtained in the one-pot reductive amination of aldehydes with nitroarenes catalyzed by heterogeneous metal-based systems of the last two decades, it is possible to draw the following conclusions. Generally, systems based on noble metals (Pd, Pt, Rh, Ir) are active under relatively mild conditions (some of them work well at room temperature, under atmospheric pressure), while the Earth-abundant metals Co and Fe, although very interesting for an economic and natural source conservation points of view, require high temperatures and pressure to be effective. On the contrary, the earth abundant Ni metal allows the design of catalytic systems that work under mild conditions, thus mimicking the performance of the noble metal catalysts.

In most cases, the heterogenous catalysts used in RA are nanostructured and for these examples the role of the support is of crucial importance. In fact, the used matrix (which can be organic or inorganic) stabilizes the metal or the metal oxide nanoparticles preventing their aggregation and allowing their recovery and recycle. For metals with low activity in hydrogenation reactions, such as Co or Fe, good results are achieved when the support is N-doped because nitrogen centers enhance the electronic features of the adjacent metals, facilitating the formation of the catalytically active metal-hydride species. At the same time, the N-doped support acts as source of Brønsted base sites, promoting the dissociation of the reducing agent, such as formic acid, and the heterolytic cleavage of dihydrogen, when the latter is used as the reductant. For these catalytic systems, in many cases the rate determining step is the nitroarene reduction, therefore, electron-withdrawing substituents in the nitroarene ring improve the final yield in the target molecule (secondary amine). However, there are few examples reporting the imine hydrogenation, as the rate determining step of the catalytic cycle. In these cases, the final yield is negatively influenced by the presence of substituents in *ortho*- position of the benzaldehyde and/or nitroarene ring. On the contrary, noble metal-based systems are very active in hydrogenation reactions, therefore the rate determining step of their catalytic cycle is usually the condensation between aniline and aldehyde. For this reason, these metals work well with supports endowed with acid functionalities, such as zeolite or organic polymers with pendant carboxylic groups, to shift the imine formation equilibrium towards the product. In these systems, electron-donating substituents on the nitroarene ring accelerate the overall reaction rate. Ni based systems are very similar to noble metal ones, but the nitroarene hydrogenation follows a different kinetic pathway, thus potentially could give more side-products. Among noble metals, Au systems are the most selective, tolerating a plethora of reducible groups on the starting reagents, while Pd systems are the most active and studied. Due to their expensive costs, many efforts have been developed in trying to easily recover and recycle the precious metal catalysts in RA. A method followed by many researchers consists in synthetize metal/magnetite nanocomposites easy to be recovered thanks to their magnetic features.

However, the most active catalytic systems sometimes have the drawback to easily hydrogenate the aldehyde before it condenses with aniline. In these cases, the RA in one-pot stepwise fashion is mandatory, with the disadvantages to carry out the one-pot tandem reactions in three steps instead of only one, as it generally occurs.

In addition, the use of water or aqueous solvents in RA is still rare, because water shifts the imine formation equilibrium towards the reagents. This thermodynamic behavior is a drawback from the sustainability point of view.

Finally, very recently some interesting examples using Au, Ag or Cu based DRA under continuous flow conditions have been developed, although they present the disadvantage to be regenerated after a while, due to deposition of carbonaceous impurities on the catalyst impregned cartridge.

Last, but not least, some heterobimetallic catalytic systems have been designed aiming at conjugating the properties of two different metals, hoping in a synergic action. However, in the reported examples, the developed catalysts seemed to follow the features of the most active metal among those employed.
